# Legume seed system performance in sub-Saharan Africa: barriers, opportunities, and scaling options. A review

**DOI:** 10.1007/s13593-024-00956-6

**Published:** 2024-03-26

**Authors:** Caitlin Breen, Noel Ndlovu, Peter C. McKeown, Charles Spillane

**Affiliations:** Agriculture & Bioeconomy Research Centre, Ryan Institute, University of Galway, University Road, Galway, H91 REW4 Ireland

**Keywords:** Legumes, Seed systems, Scaling, Smallholders, Sub-Saharan Africa

## Abstract

As a fundamental pillar of food security in sub-Saharan Africa (SSA), ensuring seed security is critical to empowering farmers in cultivating food and livestock feed, thereby fostering income generation from agricultural outputs. Among the crops cultivated by smallholders, legumes have the potential to deliver multifaceted benefits. Legumes are nutrient-dense and enhance soil health through their nitrogen-fixing qualities. However, in many instances, the development, release, and supply of improved legume varieties are insufficient to meet the needs of smallholder farmers in SSA. Here, we systematically reviewed the literature to (i) identify and categorize existing legume seed systems, (ii) map legume varieties available to smallholders, (iii) identify barriers hindering the adoption of various legume varieties, and (iv) identify potential strategies and opportunities for strengthening legume seed systems in SSA. Our results demonstrate the coexistence of formal and informal seed systems within legume seed supply chains in SSA, each employing unique seed distribution channels. Smallholders, however, are shown to predominantly depend on the informal seed system to source most legume seeds except for commercially available varieties. We also identified a diverse range of legume varieties available to smallholders in the region, with farmers having varying trait preferences based on crop type and gender. Notably, high yield and abiotic stress tolerance were the most preferred traits. The adoption of these varieties, however, is influenced by various factors, including lack of timely access to seeds in adequate quantities from the formal seed system, high seed costs, and limited information on new varieties. The reviewed literature highlighted that utilizing improved legume varieties had a positive effect on smallholders, leading to improved welfare, food security, dietary diversity, and income. We conclude that the effective scaling of legume systems in SSA is contingent upon the presence of supportive policy frameworks and well-established technical support structures.

**Graphical Abstract:**

Packets of legume seeds within a legume germplasm and breeding program at the University of Zambia (Photo by Caitlin Breen, 2022). 
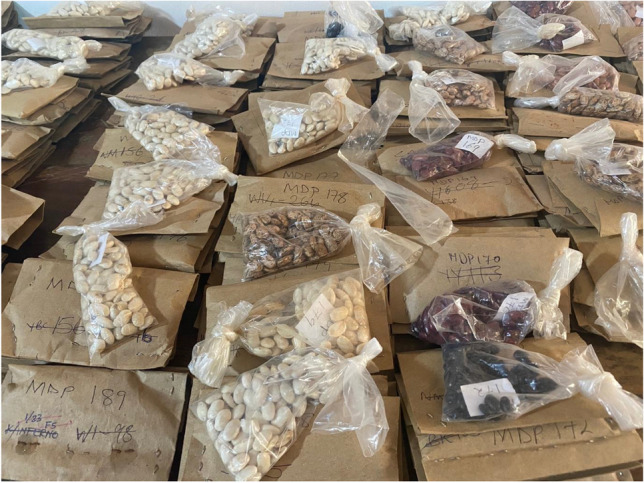

**Supplementary Information:**

The online version contains supplementary material available at 10.1007/s13593-024-00956-6.

## Contents

- 1. Introduction
- 2. Methodology
- 2.1 Search strategy
- 2.2 Literature screening and validity assessment
- 3. Results and discussion
- 3.1 Characterization of reviewed studies
- 3.2 Key stakeholders in legume seed systems across SSA
- 3.3 Informal seed networks drive legume diffusion among smallholders
- 3.4 Farmer trait preferences and available legume varieties
- 3.5 Challenges in the adoption of legume varieties and attainable adoption benefits in SSA
- 3.6 Enabling support for strengthening legume seed systems in SSA
- 4. Conclusion
- Author declarations
- References

## Introduction

Access to improved legume seed is a key element in assuring food security and climate resilience for smallholder farmers in sub-Saharan Africa (SSA). Legumes have significant benefits for use in smallholder farming systems in SSA, often being touted as “climate-smart crops” (Dutta et al. 2022) for the role they play in enhancing agricultural resilience in the face of climate change. Most importantly, their nitrogen-fixing ability facilitates the development of more sustainable cropping systems (Stagnari et al. 2017). The nitrogen-fixing ability of legumes holds a significant value for smallholder farms in SSA, characterized by nutrient-deficient and degraded soils (Kamanga et al. 2014; Kimutai et al. 2023; Ndlovu et al. 2022), especially considering the limited use of inorganic fertilizers due to their high prices. In addition to their agroecological benefits, legumes are an important nutrient-dense food staple item and are considered beneficial for both human health and the health of the planet (Aiking 2011; Foyer et al. 2016; Kumar and Pandey 2020; Shitta et al. 2021; Stagnari et al. 2017). Legumes are high in protein, important minerals (P, Ca, K, Mg, Fe, Zn, Cu, Mn, and Se), vitamins (B1, B2, B3, B5, B6, choline, E, and folate), and fiber contents (Ojiewo et al. 2015; Snapp et al. 2019). In this regard, the upscaling of legume variety adoption among smallholder farmers could serve as a pivotal factor in enhancing household food and nutrition security (Asfaw et al. 2012; Larochelle and Alwang 2022). However, despite the known benefits of legumes for both human nutrition and cropping systems, the legume seed supply systems in SSA remain underdeveloped (Rubyogo et al. 2019), particularly in comparison to well-established seed systems for major cereals like maize.

Legume seed systems in SSA operate both formally and informally, with intersections and interactions between both modes of seed delivery and access for smallholders. In most cases, formal and informal seed systems tend to exist in parallel, where the extent to which a smallholder farmer uses each system can differ depending on their geographical location (e.g., core vs peripheral region), size of the farm, cropping system, their purchasing power, social networks, ethnicity, and gender, among other factors. For most legumes, except for cash legume crops (such as soybean and common bean), smallholders rely on the informal seed system. Drawing from surveys involving more than 2500 smallholders in SSA (Malawi, Kenya, Democratic Republic of Congo, South Sudan, and Zimbabwe), McGuire and Sperling (2016) and Sperling et al. (2021) estimated that 90% of legumes are grown using seed sourced through informal seed systems. The reliance of smallholders on the informal system for their seed supplies can occur by design or default. In the former case, it arises because of inherent attributes of the informal seed system, while in the latter case, it can arise because the formal system does not provide adequate seed supply for the farmers.

Strengthening and “design for synergy” of the legume seed systems has the potential to enhance food and seed security for smallholder farmers. However, it is considered necessary to build upon the strengths of the informal seed system, without placing undue pressure on it (Wattnem 2016). Most importantly, farmer preferences can inform formal breeding programs, to help develop varieties that are preferable to smallholders as they can be high-yielding while also retaining some of the preferred traits of the local varieties such as taste or adaptation to certain soils. While significant farmer participation may not be present in all breeding programs, there have been several initiatives in SSA that involved farmers in legume variety development (Ceccarelli and Grando 2020). Despite this, a notable gap exists in the market for affordable improved legume varieties tailored for smallholder farmers. Furthermore, investments for intensifying efforts in legume breeding and enhancing the legume seed supply are currently inadequate.

Multiple reasons are cited as to why legume seed systems have not seen as much private sector investment as other crops. One reason mentioned in multiple studies is that most legume crops are self-pollinating (McGuire and Sperling 2016; Rubyogo et al. 2010; Tripp et al. 2007), similar to rice. Self-pollinated crops breed “true” where the saved seed will recapitulate the same traits as the parental lines from which the seed was collected (assuming the seed saved is not diseased and has been stored properly to maintain seed viability). Saving the seed of a proportion of the legumes planted can be used as a basis for generating seeds for the next planting season. This self-sufficiency reduces the demand for purchasing seeds each planting season, diminishing the potential market for private seed companies. While farmer-saved seed is important and has a strong intuitive appeal, it must be recognized that there are labor, capital, and “opportunity cost” inputs associated with seed saving and challenges in harvesting and storage of farmer-saved seed that maintain high germination rates and are clean (phytosanitary consideration) of pests and diseases that can suppress yields or decrease palatability. There are also significant differences between crop species, where seed saving by farmers can be more effective in some crops compared to others due to the differences in seed biology, longevity, and perishability between different crops. Furthermore, the nature of the smallholder legume market (i.e., the limited purchasing power of smallholders, costly distribution channels, diversity of legume trait needs, the reproductive biology of the crop, and lack of return on private investment) constrains legume variety development and seed dissemination via the formal system. This contrasts with the major cereal staples: maize and rice.

**Fig. 1:**
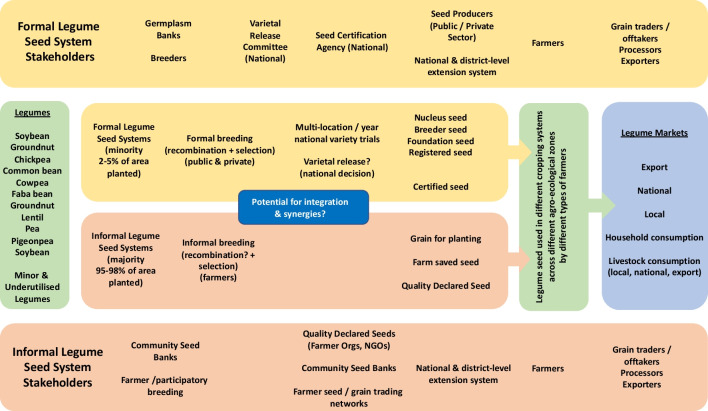
Schematic overview of stakeholders, components, and processes within formal and informal legume seed systems in SSA. In the region, the area planted to legume seeds from the informal seed systems vastly exceeds (e.g., estimated 95–98%) the area planted to legume seeds from the formal legume seed system (Chris Ojiewo, CIMMYT, pers. comm). Within the formal legume seed system, professional plant breeding involves the generation of genetic recombinants by crosses conducted by plant breeders to generate new varieties—it is unclear whether (or what) varieties in the informal system have arisen from deliberate crossing to generate new lines with new traits. However, farmers are likely engaged in the selection and identification of varieties of interest to them from available portfolios of germplasm or varieties. Within the informal seed system (and depending on legume species), farmers may plant legume seeds that span a continuum from quality declared seed, farm-saved seed, to planting of grain that has sufficient germination rates to allow a harvest to be generated. One of the biggest challenges faced in legume seed systems in the region is the lack of guaranteed or reliable markets for seed producers and farmers to make decisions to invest in producing and planting high-quality legume seed. The blue box raises the issue of what potential there is for greater integration and synergies between the formal and informal legume seed systems.

Given the current and strategic importance of legumes to smallholder farming systems in SSA, the objectives of this systematic review were to (i) identify and categorize existing legume seed systems, (ii) map legume varieties available to smallholders, (iii) identify barriers hindering the adoption of various legume varieties, and (iv) identify potential strategies and opportunities for strengthening legume seed systems in SSA which have been proposed to date. We achieved our objectives by conducting a thorough literature review and subsequently performing an empirical analysis, utilizing existing research to inform our study and derive meaningful insights on legume seed systems in SSA. This study is intended to inform ongoing work aimed at strengthening legume seed supply systems by identifying areas that require development and key actors who should be engaged in the strengthening of legume seed systems (Fig. 1).

## Methodology

A systematic literature review on legume seed systems in SSA was conducted. Prior to beginning the review, a protocol was developed for the search strategy, screening, exclusion/inclusion, and data analysis to reduce bias. Databases used in the search were selected based on a preliminary search using keywords, and those giving the highest number of results were chosen. The process was recorded and reported using a Preferred Reporting Items for Systematic Review and Meta-analysis (PRISMA) flowchart (Moher et al. 2015). PRISMA provides a standard structure for researchers to report reviews and meta-analyses in a transparent way (Sarkis-Onofre et al. 2021).

### Search strategy

The electronic databases (1) Web of Science, (2) Science Direct, (3) PubMed Central, (4) ProQuest, and (5) Google Scholar were chosen to search for peer-reviewed research articles published from 2002–2022 (last search on 30th of May 2022). Key search terms were identified before beginning the advanced search. These include but are not limited to:

(i) (Legumes OR pulses) AND (seed OR varieties) AND (sub-Saharan Africa OR Africa)
(ii) (Seed systems OR seed system) AND (formal and informal) AND (seed policies OR legislature) AND (stakeholders) AND (sub-Saharan Africa OR Africa)
(iii) (Scaling OR scale) AND (barriers OR constraints) AND (legume seed OR seed systems) AND (sub-Saharan Africa OR Africa)

The number of results (retrieved papers) obtained from each search was documented. Three searches were performed within each of the 5 databases (the first search was carried out on the 16th of May 2022 and the final search on the 30th of May 2022). The first search focused on “legume seed(s)” in SSA, the second one looked at existing “legume seed systems and policies/regulatory frameworks,” and the last search was on “options” and “barriers” to scaling legume seed systems in SSA. A range of search terms and combinations of Boolean operators were used. The search queries used in each database are presented in Table 1.

**Table 1:** Search queries used in each database.

	Web of Science	Science Direct	PubMed Central	ProQuest	Google Scholar
Search 1	ALL= ((“legume” OR “legume seed” OR “pulses” OR “legume varieties) AND (“sub-Saharan Africa” OR “SSA” OR “Africa”)) Year = 2002–2022	(“legumes” OR “legume seed” OR “legume varieties) AND (“sub-Saharan Africa” OR “SSA” OR “Africa”) Year = 2002–2022	(((“legume” OR “legume seed” OR “pulses” OR “legume varieties”))) AND ((“sub-Saharan Africa” OR “SSA”)) Year = 2002–2022	(Legume seed OR legume) AND (sub-Saharan Africa OR SSA)	“legume” “seed” “Africa” “sub-Saharan Africa” “legume seed”
Search 2	ALL= ((“seed systems” OR “legume seed systems” OR “seed policy*” OR “legislature” OR “stakeholders” OR “formal or informal”) AND (“sub-Saharan Africa” OR “SSA” OR “Africa” ))	(“legume seed systems” OR “seed systems” OR “seed policy” OR “legislature” OR “formal or informal”) AND (“seed”) AND (“sub-Saharan Africa” OR “SSA” OR “Africa”)	((“seed systems” OR “seed policy*” OR “legislature” OR “formal or informal”) AND (“sub-Saharan Africa” OR “SSA” OR “Africa”)) Year = 2002–2022	(“seed systems” OR “seed policy*” OR “legislature” OR “stakeholders” OR “formal or informal”) AND (“legumes”) AND (“sub-Saharan Africa” OR “SSA” OR “Africa”)	“seed” “legumes” “Africa” “seed systems” “legume seed systems” “sub–Saharan Africa”
Search 3	ALL= ((“scaling” OR “scale” OR “barriers” OR “constraints” OR “options” OR “opportunities” OR “Possibilities” OR “Feasibility” OR “Prospects” OR “Difficulties” OR “Problems”) AND (“legume seed” OR “seed systems” OR “legumes*”) AND (“sub-Saharan Africa” OR “Africa” OR “Tanzania” OR “Ethiopia” OR “Malawi” OR “Zambia”))	(“scaling” OR “barriers” OR “options” OR “Possibilities” OR “Problems”) AND (“legume seed” OR “seed systems”) AND (“sub-Saharan Africa” OR “Africa”) (“scale” OR “constraints” OR “opportunities” OR “Feasibility” OR “Prospects” OR “Difficulties”) AND (“legume seed” OR “seed systems”) AND (“Africa”)	(“scaling” OR “barriers” OR “scale” OR “constraints” OR “opportunities” OR “Feasibility” OR “Prospects” OR “Difficulties” OR “options” OR “Possibilities” OR “Problems”) AND (“legume seed” OR “seed systems”) AND (“sub-Saharan Africa” OR “SSA” OR “Africa” OR “Tanzania” OR “Ethiopia” OR “Malawi” OR “Zambia”)	(“scaling” OR “barriers” OR “scale” OR “constraints” OR “opportunities” OR “Feasibility” OR “Prospects” OR “Difficulties” OR “options” OR “Possibilities” OR “Problems”) AND (“legume seed” OR “seed systems”) AND (“sub-Saharan Africa” OR “SSA” OR “Africa” OR “Tanzania” OR “Ethiopia” OR “Malawi” OR “Zambia”)	“scaling” “legumes” “barriers” OR “options” OR “constraints” OR “opportunities” OR “scale” “seed systems” “legume seed”

### Literature screening and validity assessment

Following the initial search, the title and abstract of each article were screened. Documents meeting inclusion criteria for this systematic review were peer-reviewed research articles that reported on various aspects related to legume seed systems, smallholder legume farmers, barriers to the adoption of improved varieties, outcomes of successful legume variety adoption, and opportunities for strengthening existing legume seed systems. Additionally, inclusion was limited to papers focusing on sub-Saharan Africa (SSA). We also excluded non-peer-reviewed publications such as pamphlets, reports, proceedings, or any publications not in the English language. Using this approach, the relevant subset of papers was then extracted. Papers were excluded through a screening process involving the evaluation of titles and abstracts, as well as the removal of duplicate entries. This process was performed for each database. Articles were exported to EndNote 20 (Clarivate Analytics) to identify and remove duplicates. The removal of duplicates was done using the “Find Duplicates” function of the EndNote referencing software. Any duplicates not identified by the software were removed manually when encountered.

Screening of the full-text of each peer-reviewed article was then performed by reading the paper and identifying the study design, key findings, and recommendations of the paper (Supplementary Materials). Papers found not to be relevant (i.e., not based in SSA, based outside timeframe (2002–2022) and findings not related to legume seed systems) at this point were excluded. Papers of the wrong type (i.e., review articles, reports, discussion papers, pamphlets, proceedings, bulletins) were also excluded. The number of articles excluded based on full-text screening was documented. Data and findings were extracted from the full-text articles and inserted into tables. The final number of papers used in this review was presented on the PRISMA flowchart. Supplementary Tables 2, 3, 4, 5, 6, and 7 display the study design, key findings, and recommendations of all papers used in the review. The Joanna Briggs Institute (JBI) Critical Appraisal Tool (Munn et al. 2014) was used for quality control.

## Results and discussion

Seed security is essential in achieving food and nutrition security in SSA. Presently, smallholder farmers source their legume seeds predominantly from the informal seed system, with some smallholders having access to legume seeds from the formal system. However, access to improved legume varieties is inconsistent and unevenly distributed. In SSA, both the formal and informal seed systems are vulnerable to disruptions (such as climate change, economic shocks, the COVID-19 pandemic, and conflicts), leaving smallholders at risk of both seed and food insecurity. Strengthening the resilience of legume seed systems that serve smallholder farmers is, therefore, critical for implementing effective adaptation measures within the SSA agricultural sector. Our systematic review analyzes the barriers to legume seed adoption and options/opportunities for scaling the efficiency of existing legume systems in SSA.

### Characterization of reviewed studies

**Fig. 2:**
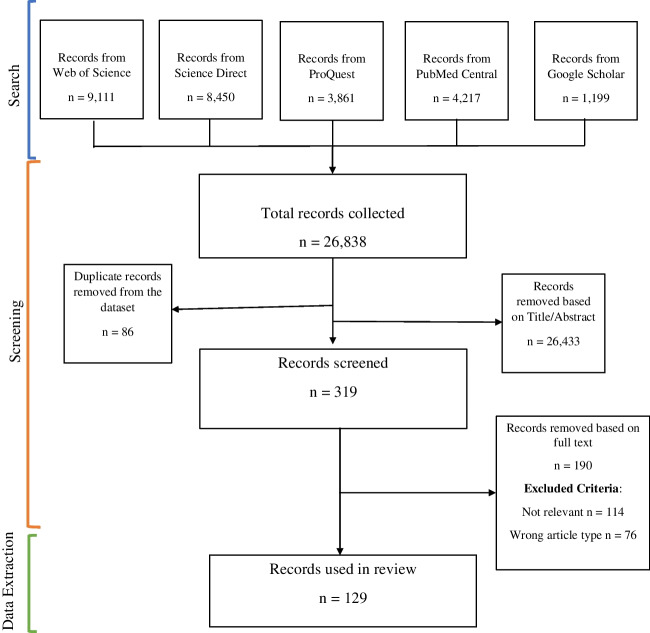
PRISMA flow diagram of search strategy, literature screening, and validity assessment. A total of 129 research articles were retrieved from 5 databases for systematic review.

In total, searches across 5 databases using the search strings outlined in Table 1 yielded 26,838 results (Web of Science, *n* = 9111, Science Direct, *n* = 8450, ProQuest, *n* = 3861, PubMed Central, *n* = 4217, and Google Scholar *n* = 1199). Following title and abstract screening based on predefined criteria, 26,433 results were excluded, leaving 405 studies for further analysis. An additional 86 duplicates were identified and removed. A total of 319 papers were initially screened using the search strategy outlined in Fig. 2. After a thorough full-text screening, 190 papers were excluded: 58% (*n* = 114) were deemed irrelevant, and 42% (*n* = 76) were of an incorrect article type (e.g., review papers, reports, or conference papers). This process resulted in 129 papers being included in the final systematic review. Specifically, in the scope of the reviewed articles (*n* = 129), 28% (*n* = 36) focused on the current status of seed systems in SSA (Supplementary Table 2). More than half (*n* = 18) of these 36 papers analyzed seed systems generally, 2 papers examined the operating policy mechanisms in seed systems, 12 articles looked at local seed exchanges/seed networks, and 4 papers reported on seed security.

**Fig. 3:**
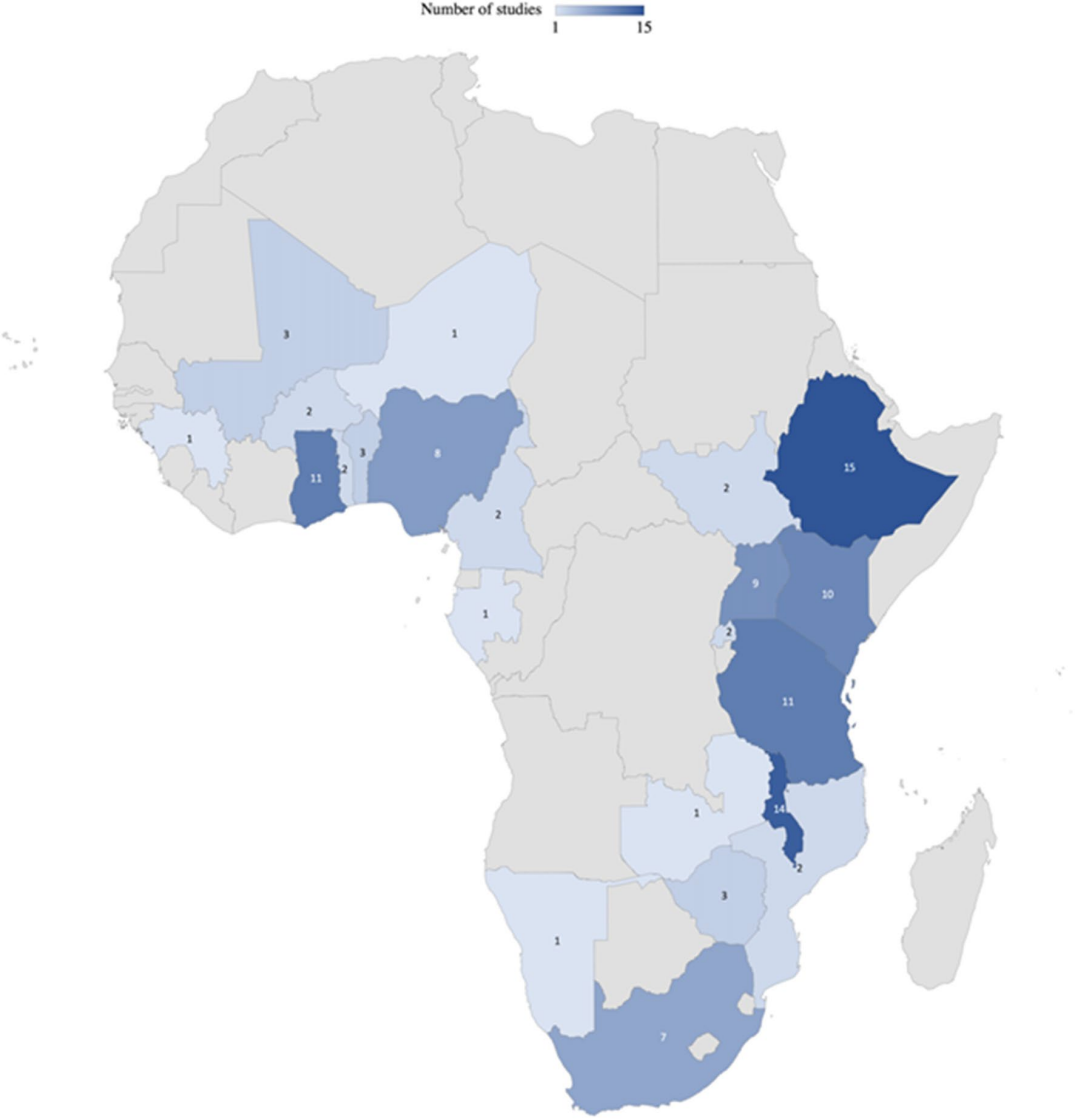
The map of Africa highlights countries that were the primary focus of one or more legume seed system studies in the systematic review, with the numbers denoting the number of studies for each respective country. Regional studies, encompassing more than one country (*n* = 18), are excluded from this representation. Countries without any focus in this review are shaded in gray.

Figure 3 illustrates the primary countries of focus in the reviewed research articles. The predominant focus within SSA was Ethiopia (*n* = 15), closely followed by Malawi with 14 research articles. Conversely, only one study was identified in Zambia, Namibia, Gabon, Niger, and Guinea. The distribution of research work on legume seed systems indicates a concentration of studies in Eastern Africa based on our findings

### Key stakeholders in legume seed systems across SSA

Our findings indicate that seed systems in SSA are characterized by a combination of formal and informal structures, with some evidence pointing to integrated systems that amalgamate elements from both systems. To some extent, each of these systems depends on the other to operate, and there are connections between both systems. The connections between formal and informal legume seed systems in SSA materialize as farmers procure improved crop varieties, often on a small scale (e.g., for soybean and common bean), through purchases or seed aid/input support schemes, preserving them for future use within their traditional informal seed systems. Furthermore, informal seed systems can act as reservoirs of diversity and resilience, providing locally adapted traditional varieties that complement the formal system and enhance overall seed system sustainability and resilience. These legume seed systems in SSA are characterized by a diverse range of stakeholders operating at various levels and across multiple legume crops.

In this region, the formal seed system usually includes government entities such as Ministries of Agriculture, National Agriculture Research Systems (NARS), professional breeding programs, varietal release and seed certification bodies, national extension systems, and seed companies (McGuire and Sperling 2016; Alemu 2015; Wattnem 2016). The informal seed system, on the other hand, typically involves the distribution and exchange of seed or planting materials outside of the formal seed system. This encompasses seeds of varieties (registered or unregistered, including landraces) saved and used by farmers or informal rural entrepreneurs. Our analysis identified farmers, traders, NGOs, extension agents, cooperatives, and companies (e.g., seed companies or agro-dealers) (Ayenan et al. 2017b; Branca et al. 2021) as key stakeholders in both the formal and informal legume seed systems. Generally, our analysis revealed that formal seed systems are characterized by the involvement of stakeholders such as seed companies, government agencies, and research institutions, while informal seed systems are predominantly constituted by smallholder farmers, community seed banks, and local markets.

Some stakeholders in the seed system, such as genebanks, breeding programs, and seed quality control and certification agencies, were not prominently emphasized in the literature reviewed. Nevertheless, they were encompassed in the seed system mapping diagram depicted in Fig. 1, underscoring their roles within the system. However, it is noteworthy that none of the studies in the review primarily focused on seed system stakeholders. We also identified agro-dealers and farmers’ organizations as crucial in linking the formal and informal seed systems. However, there is a knowledge gap within the literature surrounding the needs of key stakeholders and value chain actors across legume seed systems. This area could be researched further to assist in strengthening legume seed systems for the future. Our results also indicated that legume seed distribution in the formal sector is done mainly by agro-dealers, seed companies, and cooperatives. Informal channels for accessing seed, on the other hand, include farmers’ own saved seed stocks, local markets, and social networks such as exchanges with other farmers or gifts (Sperling et al. 2021). The seed policy mechanisms in SSA, however, were reported to be more concentrated on all system processes (i.e., seed production, processing, and distribution) of the formal seed system. In addition, most operating seed regulatory frameworks in SSA do not recognize informal seed production and distribution channels.

### Informal seed networks drive legume diffusion among smallholders

Our results indicated that the informal system is the primary source of legume seed for the vast majority of smallholders in the region (Mulesa et al. 2021; Ayenan et al. 2017b; Kilwinger et al. 2021; Marimo et al. 2021; McGuire and Sperling 2016; Nordhagen and Pascual 2013). It is, however, essential to underscore that this scope excludes cash legumes (e.g., soybean and common bean), as their seed supply is primarily controlled by the formal sector and influenced by market demand, government policies, and interest from research institutes. The dominance of the informal seed sector has been documented in a range of reports focusing on SSA (Rubyogo et al. 2016; McGuire and Sperling 2016; Sperling et al. 2020). For instance, according to Alemu (2015), during the 2009/2010 cropping season (as per Central Statistical Agency data), approximately 84.75% of the cultivated land for all crops in Ethiopia was sown using locally sourced seed. What is not clear are the reasons for the predominant reliance of smallholders on the informal seed system for legume seed supply. It is most likely that such reliance arises from a lack of access by smallholders to improved varieties from the formal seed system. This has been acknowledged by several studies (van Niekerk and Wynberg 2017; Nordhagen and Pascual 2013), emphasizing that informal seed systems act as a safety net during periods of shocks or disasters, especially when the formal system falls short of meeting seed demands. Given the choice of accessing legume seeds from formal and informal systems, it remains unclear whether smallholders would choose to source from one or indeed from both systems. While it has been shown that smallholders use more locally sourced varieties than improved varieties, particularly for legumes, the barriers to accessing improved legume seeds remain a key challenge (Snapp et al. 2019). However, this applied mostly to legumes that are commonly grown in smallholder farmer communities (such as cowpea, Bambara nut, and groundnut).

One common factor (and challenge) across the informal seed system is that there is no legal certification requirement regarding the quality (e.g., germination rate, identity) of the seed supplied or exchanged (Sisay et al. 2017). Despite this, the informal system (where it is functioning) can provide farmers with seed at the time and place they require it, when seed cannot (for whatever reason) be accessed through the formal system. Conversely, situations could arise where the informal seed supply system is constrained (e.g., during a lean period or conflict when seed stocks may have to be consumed), and formal seed systems may be necessary for the provision of replacement of planting materials. While local seed markets are considered by some commentators to be a more flexible, sustainable, and reliable way to source seed (Sisay et al. 2017; Alemu 2015), the resilience of both formal and informal seed systems to shocks needs to be considered. Indeed, a more integrated formal and informal seed system may serve as a more resilient system, compared to either system on its own.

Our findings also showed that legume seed access via the informal seed system in SSA is influenced by a range of factors. These include infrastructure (i.e., roads to access rural areas), gender inequality, and access to credit facilities (Mulesa et al. 2021; de Boef et al. 2021; Madin et al. 2022). Social capital (i.e., relationship networks) was also seen to be very important in accessing seed informally (Ricciardi 2015). Sisay et al. (2017) highlighted that while some of the varieties exchanged between farmers will be “local” varieties (e.g., landraces), others will be “improved” varieties that have previously been obtained through the formal system. On the patterns of legume seed access, our findings showed this to be influenced by farmers’ social networks, marriage systems (matrilineal vs patrilineal), and ethnicity (Almekinders et al. 2020; Delêtre et al. 2011; Labeyrie et al. 2016). For example, some studies suggest that female farmers depend to a greater extent on their social networks than male farmers, as they may have less access to key actors within the formal system, such as agro-dealers (Marimo et al. 2021; Otieno et al. 2021). It is considered important to strengthen connections and enhance collaboration between the formal and informal seed systems (Kuhlmann and  Dey 2021; Ayenan et al. 2017b). Although the results of this study show that the informal seed system can be resilient in the face of shocks and can provide seed following crisis periods (van Niekerk and Wynberg 2017; Nordhagen and Pascual 2013), the informal system is not inherently reliable as a consistent source of improved seed. In addition, while social capital can be considered an asset, it is important to consider how marginalized individuals or groups that lack social capital can as a result have more limited access to seeds from an informal seed system (Cleaver 2005).

In principle, informal seed systems can be a less expensive way of accessing seed, in situations when seed costs (and possibly profit margins) are lower than what seed companies are charging or where farmers can purchase seed in smaller quantities. Access to smaller unit quantities of seed can allow farmers to sow a smaller piece of land or to test out a crop without having to commit to the scale of bags supplied by some seed companies or large-scale seed programs (Sisay et al. 2017; Alemu 2015; Rubyogo et al. 2016). The decentralized nature of the informal system is also an important consideration as it can mean that farmers in peripheral rural areas can potentially have local access to seed, which they would not have if they depended solely on any formal system that is unnecessarily centralized. Due to its unregulated nature and lack of formal investment, the perception that the informal seed system can operate reliably and consistently over time, geography, and communities may not be true and needs to be considered in relation to resilience, food, and livelihood security. There are also risks of fraud that need to be considered, e.g., through the sale of seeds that are not the varieties claimed or of seeds with low germination rates. Indeed, the promotion of a fully decentralized informal seed system where farmers and their communities are expected to finance and produce their seed can be seen as a form of “rolling back the state” where the responsibilities and roles for seed production, quality control, and dissemination are no longer a responsibility of government (Cook et al. 2021).

Despite the benefits that the informal system has for smallholder farmers, there are also challenges associated with the informal system (Snapp et al. 2019). The yield potential goes down each year when farmers reuse saved seeds or recycled seeds from the informal seed system. Furthermore, the informal seed systems are not necessarily considered an efficient mechanism for the distribution of new varieties (McGuire and Sperling 2016) and can lack the capacity to deliver both the quality and quantity of seed needed (Shiferaw et al. 2008). In this respect, improved coordination of legume seed systems is urgently needed as neither the informal nor the formal system is fully meeting the seed demands of farmers in SSA. A closer look at the operating legume seed systems indicated that there are some connections between the formal and informal seed systems, which should be further strengthened (Kuhlmann and Bhramar 2021; Ayenan et al. 2017b) to ensure legume seed security in SSA.

### Farmer trait preferences and available legume varieties

Legume varieties encompass both formally released varieties, which have undergone multi-locational yield trials at the national level before being recommended for release to farmers, as well as those accessible through the informal system, obtained via purchase, exchange, or farmer-saved seed. The latter can also include “landrace” varieties. Irrespective of the source of the variety, the litmus test for consistent farmer adoption of any legume variety will be its performance in the farmer’s field and the marketability of its produce (whether for household consumption and/or income). Hence, a focus on specific legume varieties is important for studies to be relevant to smallholder farmers’ lived realities. Over the years, legume breeding programs in SSA have released several legume varieties conferring improved climate resilience, nutrition, and yielding capacity (Varshney et al. 2019). Such released varieties have been selected for desirable traits and should have undergone rigorous testing and trials to ensure their quality before they are commercialized. However, there are also “local” varieties or landraces grown by farmers which may have never been registered and are saved by farmers. It is possible that some locally “named” varieties may have originated as registered seeds purchased by a farmer or obtained from an input scheme. Regardless of the source of the seed, farmers choose to grow varieties that have traits they desire and which they can access. In this respect, seed system innovations are needed to ensure that new legume traits and varieties reach smallholder farmers (Westengen et al. 2023).

In our study, only 15% (*n* = 19) of the total reviewed papers listed the names of legume varieties used in their studies (Supplementary Table 3). Of these, only 10% (*n* = 2) discussed legume varieties generally, while 42% (*n* = 8) listed legume traits preferred by farmers, and 53% (*n* = 9) specified particular legume variety names. Our study identified a greater number of released groundnut varieties (*n* = 5 (29%) than any other crop, followed by cowpea. This may be due to greater market demands for groundnut or due to efforts to reduce losses faced by farmers because of aflatoxin contamination. Multiple papers reported on common bean (*n* = 3) and cowpea, Bambara nut, and pigeonpea (with two papers each). Soybean, navy bean, lablab, faba bean, and garden pea were each the subject of a single study. Overall, 28 legume varieties were identified by our review study (Table 2): groundnut (*n* = 11), common bean (*n* = 4), pigeonpea (*n* = 5), and cowpea (*n* = 8). Most (*n* = 12) of the identified legume varieties are available for farmers in Tanzania. Groundnut varieties, *BaHajidu*, *Bulki-01*, *Werer-963*, and *Werer-963* are available for farmers in Ethiopia, with *BaHajidu* being the highest yielder. Also available in Ethiopia are the common bean varieties, *Nasir* and *Goberesha*. In Tanzania, five pigeonpea varieties (*ICP 7035*, *ICPL 90094*, *Kat 50*, *QP37*, and *ICP 86005*) were reported to be available to farmers along with the six groundnut varieties (*Johari 1985*, *Pendo 1998*, *Naliendele 2009*, *Mnanje 2009*, *Mangaka 2009*, and *Nachi 2015*). In Nigeria, four cowpea varieties, *IT8ID-699*, *TVx3236*, *IT82E-18*, and *IT84S-2246-4*, were identified in our analysis. For studies focusing on South Sudan, four cowpea varieties were identified: *IT90K-277-2*, *ACC004*, *IT07K-211-1-8*, and *Mading Bor II*. In South Africa and Kenya, the *ICGV 03796* (groundnut) and *Nyota* (common bean) varieties are available on the market. The literature we reviewed also demonstrated that the absence of any of these varieties in the market at the beginning of the agricultural season can hinder the uptake of these varieties (Nchanji et al. 2021b; Mwalongo et al. 2020).

**Table 2:** Released legume crop varieties in sub-Saharan African countries.

Legume crop	Variety name	Country	References
Groundnut	*BaHajidu*, *Bulki-01*, *Werer-963*,* Werer-963*	Ethiopia	Belayneh and Chondie (2022)
	*Johari 1985*, *Pendo 1998*, *Naliendele 2009*, *Mnanje 2009*, *Mangaka 2009*,* Nachi 2015*	Tanzania	Mwalongo et al. (2020)
	*ICGV 03796*	South Africa	Hoffmann et al. (2018)
Common bean	*Nasir*,* Goberesha*	Ethiopia	Merga (2020)
	*Nyota*	Kenya	Nchanji et al. (2021b)
	*Lyamungu 90*	Tanzania	David et al. (2002)
Cowpea	*IT8ID-699*, *TVx3236*, *IT82E-18*,* IT84S-2246-4*	Nigeria	Giami (2005)
	*IT90K-277-2*, *ACC004*, *IT07K-211-1-8*,* Mading Bor II*	South Sudan	Ngalamu et al. (2020)
Pigeonpea	*ICP 7035*, *ICPL 90094*, *Kat 50*, *QP37*,* ICP 86005*	Tanzania	Mligo and Craufurd (2005)

Based on a number of breeding program pipelines, a range of legume varieties are being developed and released throughout SSA. Typically, the processes of professional plant breeding, varietal release and registration, seed certification, and supply channels of certified seed from formally released varieties constitute the formal system. Through the processes of germplasm collection, characterization, and use in breeding programs, the formal system also has a relationship with germplasm (e.g., of landraces or traditional varieties) that ultimately has its origins in the informal seed system (Wattnem 2016). The legume seed supply system for SSA is inherently connected to the breeding and varietal pipelines (whether formal or informal) that provide improved legume varieties to farmers across the region. The formal breeding programs typically combine the public sector breeding activities of the CGIAR with those of National Agricultural Research Systems (NARS), with some private sector breeding for selected legume crops (e.g., soybean and common bean). For instance, the International Center for Tropical Agriculture (CIAT) has been working closely with national programs via the Pan-Africa Bean Research Alliance (PABRA) to develop biofortified common bean varieties with high iron and zinc content (Ojiewo et al. 2015). Most of the breeding activities to develop improved legume varieties for the region are derived from CGIAR and NARS efforts and are predominantly focused on major legumes such as common bean, soybean, pigeonpea, lentil, cowpea, chickpea, and groundnut. In comparison, there are limited breeding efforts to develop improved varieties of minor and underutilized legumes such as Bambara nut, winged bean, African yam bean, and grasspea (Olanrewaju et al. 2021).

On legume traits, our results indicate that farmers generally prioritize high yield when choosing a legume variety to grow but may also consider other secondary traits (such as taste and nutritional value) in legumes. Other traits of interest include early maturity, disease/pest resistance, drought/heat stress tolerance, low labor requirements, and taste (Ayenan et al. 2017a; Mutari et al. 2021). We also found evidence indicating that while yield stood out as the primary preferred trait among both male and female farmers, other trait inclinations may be influenced by gender (Mwalongo et al. 2020; Tabe-Ojong et al. 2021). It is posited that male farmers tend to have market-oriented preferences for varieties that are affordable and accessible (Nchanji et al. 2021b; Mwalongo et al. 2020). Female farmers, on the other hand, were reported to prefer landraces or varieties with low labor requirements (Nchanji et al. 2021b). However, whether there exist specific traits unique to landraces that are favored remains unclear.

For all these crops, our analysis suggests that, for an improved legume variety to be successfully adopted in SSA, it must be high-yielding and have additional traits that match farmer preferences. A key challenge for the improvement of minor and underutilized legume crops is the market failure where the purchasing power of the consumers (i.e., smallholders and poorer rural communities) is insufficient to warrant significant investment in improvement programs for minority crops (e.g., germplasm collection, curation, crossing/breeding programs, multi-locational and multi-annual trials). Initiatives such as the African Orphan Crop Consortium are developing genomic resources and engaging in the training of young breeders that can provide a basis for the improvement of 101 African orphan crops (of which 11 are legume crops). By strengthening breeding and seed systems of minor and underutilized legume crops, the erosion of the genepool of orphan legume species can be abated, while generating new improved legume varieties of orphan legume species, which if adopted more widely can help promote resilience across farming systems.

### Challenges in the adoption of legume varieties and attainable adoption benefits in SSA

Scaling legume production in SSA is hinged on the large-scale and sustained adoption of legume varieties that smallholders consider and value as important to their farming and livelihoods (Shilomboleni et al. 2022). This significant increase in adoption by farmers must be enabled by improving the performance of the existing seed systems, both formal and informal. Our findings consistently showed that both the impacts of adopting legume varieties and the challenges hindering their adoption were recurring themes across reviewed studies (Table 4 and Supplementary Table 4). Most of the analyzed studies (67%, *n* = 86) focused on strategies to promote the widespread adoption of legume varieties, highlighting both obstacles and potential pathways for scaling up legume variety adoption among farmers. However, only one paper addressed the topic of strengthening legume seed systems, while 34% (*n* = 29) of the papers focused on the adoption of improved varieties, encompassing associated perceptions, constraints, and impacts. Barriers to the adoption of improved legume varieties were highlighted in 15% (*n* = 13) of these papers, and potential options for strengthening legume seed systems were highlighted in 48% (*n* = 41) papers (Table 3).

**Table 3:** Factors influencing the low adoption of improved legume varieties. (*x*) in the first column refers to the number of papers that mentioned this influencing factor. Factors affecting the adoption of legume varieties refer to the main factors that govern farmers’ decisions to adopt improved varieties of legumes.

Factors influencing	Crop	References
Lack of adequate finances to invest in the development of new varieties can negatively influence the availability of farmer-preferred legume varieties (1). This can also lead to slower varietal development and release – resulting in a limited number of improved legume varieties for smallholder farmers to adopt	Bambara nut, chickpea, lentil	Agyeman et al. (2021)
Lack of timely access to improved legume seed negatively influences adoption rates (7) from the formal seed system – this can be due to inefficient demand estimation mechanisms for formal seed supply	Bambara nut, pigeonpea, chickpea, common bean, lentil, climbing bean	Agyeman et al. (2021); Asfaw et al. (2012); David et al. (2002); Dessalegn et al. (2022); Mwalongo et al. (2020); Ronner et al. (2018); Shiferaw et al. (2008)
Lack of access to information about new varieties has a negative influence on adoption (4)	Pigeonpea, chickpea, soybean	Asfaw et al. (2012); Dionco-Adetayo et al. (2002); Mahama et al. (2020)
Minimal promotion of variety influences adoption negatively (1)	Common bean	David et al. (2002)
Lack of access to other agri-inputs can influence legume seed adoption negatively (2). Some legume varieties are perceived to require high fertilizer and pesticide application—this can put a strain on resource-constrained farmers leading to them not adopting such varieties	Chickpea, lentil, climbing bean	Dessalegn et al. (2022); Ronner et al. (2018)
Land ownership is a barrier to adoption (1). Farmers with smallholding often tend to prioritize staple and cash crops	Chickpea, lentil	Dessalegn et al. (2022)
Cultural norms can influence adoption (2)	Chickpea, pigeonpea	Dessalegn et al. (2022); Grabowski et al. (2019)
Poor soil fertility negatively influences adoption (1)	Navy bean	Mutari et al. (2021)
Drought/heat stress negatively influences adoption (1)	Navy bean	Mutari et al. (2021)
Farmer age positively influenced adoption (2)	Groundnut, soybean	Mwalongo et al. (2020); Mahama et al. (2020)
Adoption of different crops differed due to the farmer’s gender (1)	Groundnut	Mwalongo et al. (2020)
High seed cost negatively influences adoption (1)	Groundnut	Mwalongo et al. (2020)
High labor requirements negatively influence adoption (1)	Common bean	Nchanji et al. (2021b)

The predominant constraints identified in the reviewed literature included inadequate and untimely access to seeds in sufficient quantities from the formal seed system (*n* = 7), as well as a shortage of information regarding available improved legume cultivars (*n* = 4) (Agyeman et al. 2021; Asfaw et al. 2012; David et al. 2002; Dessalegn et al. 2022). According to Dessalegn et al. (2022), lack of access in terms of timing and quantity to improved legume seeds makes farmers rely on saved seeds. In most cases, studies have shown that there are inefficiencies in estimating or predicting farmers’ demand for legume seed and communicating this to seed suppliers to ensure a functioning seed supply value chain. The result of this is that legume seeds regularly do not arrive in time for the planting season, leading to the situation where even the willing buyers of improved seed have to depend on recycled seed or seed exchanged with other farmers (Dessalegn et al. 2022). Our review also identified high seed cost (for legumes such as common bean and soybean), inefficient demand estimation mechanisms for formal seed supply, poor quality seed from the informal, and lack of high-yielding and pest/disease resistant varieties due to poor investment in legume breeding. In some cases, cultural norms were seen to contribute significantly to adoption rates—especially for crops such as chickpea and pigeonpea. Furthermore, adoption rates for some legume crops (e.g., groundnut) were reported to differ due to the farmer’s gender. Poor soil quality in SSA was recognized as a key limiting factor to the adoption and demand of legume seeds (making the seed business unviable). Shocks such as extreme weather (affects seed production) and the COVID-19 pandemic (and its effects on seed trade) were also reported in some studies. Furthermore, it was observed that policy or regulatory mechanisms within the region impose limitations on the operation of seed systems, which in turn can affect the adoption of legumes (Ali and Awade 2019; Mulesa and Westengen 2020; Nchanji et al. 2021a).

In our analysis, we identified a small subset of papers (Supplementary Table 5) that focused on the adoption of orphan/underutilized legume varieties, rather than the more commonly cultivated legumes. These papers underscored analogous barriers to adoption and benefits associated with improved varieties, akin to the studies focusing on commonly cultivated legumes. Although this is a small number of studies, these findings demonstrate that these benefits/barriers exist for orphan/underutilized legume seed systems. As for major legume crops, most of the studies reporting on orphan/underutilized legumes reported that male and female farmers have different crop preferences.

Multiple papers reported on the impacts that the adoption of improved legume varieties has on smallholder farming communities in SSA (*n* = 11). Our findings indicate that the adoption of improved legume varieties can have positive impacts on smallholder households. While most farmers are aware that improved seed varieties are important and valuable additions to a cropping system, without access to the required quantity of new or improved seed varieties at the right time, most farmers are dependent on saved seed obtained through the informal system. The benefits of improved varieties which were highlighted in this review include improved welfare, food security, dietary diversity, and increased income (Ahmed et al. 2016; Asfaw et al. 2012; Larochelle and Alwang 2022) (Table 4). Noteworthy, positive outcomes of legume seed adoption were only associated with the fair cost of seed, farmer age (with younger farmers willing to try new varieties) and gender, and improved yield and income (Dionco-Adetayo et al. 2002; Mwalongo et al. 2020; Tufa et al. 2019).

**Table 4:** Outcomes of adoption of improved legume varieties. (*x*) in the first column refers to the number of papers that mentioned this adoption outcome.

Adoption outcomes	Crop	References
Positive impact on the welfare of farmer households (1)	Groundnut	Ahmed et al. (2016),
Reduced poverty due to increased household income (6)	Pigeonpea, chickpea, groundnut, cowpea, soybean	Asfaw et al. (2012); Konja et al. (2019); Manda et al. (2019); Shiferaw et al. (2008); Tufa et al. (2019); Verkaart et al. (2017)
Higher food security among adopting households (2)	Pigeon pea, chickpea, common bean	Asfaw et al. (2012); Larochelle and Alwang (2022)
Higher dietary diversity among adopting households (1)	Common bean	Larochelle and Alwang (2022)
Yield increase compared to non-improved legume varieties (1)	Soybean	Tufa et al. (2019)

### Enabling support for strengthening legume seed systems in SSA

The improvement of legume seed systems encompasses all the processes aimed at enhancing the production and availability of improved varieties. Our review indicated that several factors affect the successful development of existing legume seed systems in SSA (Table 5 and Supplementary Table 6). These include unequal access to extension services and credit between men and women (which can influence the total demand for improved varieties), poor soil quality, restrictive governance, and in recent years the COVID-19 pandemic (Ali and Awade 2019; Mulesa and Westengen 2020; Nchanji et al. 2021a). Lack and high cost of agricultural inputs such as fertilizers were also mentioned as barriers but less frequently than the previous barriers mentioned. In addition, anti-nutritional compounds/factors are a barrier existing in the production of some legume crops, particularly aflatoxin in relation to groundnuts (Boni et al. 2021) and ODAP (causing lathyrism) in relation to grasspea (Girma et al. 2011). Most of these constraints primarily impact production; however, their effect on the demand for legume seed has been extensively reported, impeding heightened interest from the private sector to invest in legume seed systems.

**Table 5:** Barriers to scaling legume seed systems, (each barrier was mentioned in a single study). The crop is listed as “multiple” when the barrier applies to a range of legume crops. In this study, barriers to scaling legume seed systems focused on the challenges and obstacles that limit the expansion of seed systems such as policy constraints or inadequate infrastructure.

Barrier	Legume crop	References
Poor soil quality affects the performance of some varieties thereby reducing the likelihood of adoption by smallholders	Cowpea	Anago et al. (2021)
Some legume crops/varieties have a greater susceptibility to pests and diseases resulting in lower yields. This can act as a barrier to the adoption of some legume varieties/crops by smallholders.	Cowpea	Anago et al. (2021)
Aflatoxin contamination negatively influences the cultivation of groundnuts in some smallholder farming communities	Groundnut	Boni et al. (2021)
Antinutrients in legume products can deter smallholders from producing/consuming certain legumes	Grass pea	Girma et al. (2011)
Restrictive policies can make the trade of legume seeds difficult	Multiple	Mulesa and Westengen (2020)
COVID-19 had a severe negative impact on seed trade in the region	Common bean	Nchanji and Lutomia (2021)
Lack of improved varieties (incl. early generation seed) and high seed cost	Groundnut	Sinare et al. (2021)
Limited access to land and gender issues around land tenure security (socio-economic factors)	Multiple, groundnut	Branca et al. (2021); Sinare et al. (2021)
Limited access to other agronomic inputs (such as tools and fertilizers)	Multiple	Branca et al. (2021)
Insufficient access to credit and extension services	Multiple	Branca et al. (2021)

Our study also identified a range of options for enabling the scaling of seed systems (Table 6 and Supplementary Table 7). These options focused more on social, commercial, and economic aspects such as the support and development of seed enterprises, the use of local knowledge (in the development and delivery of seed), and the encouragement and enabling of greater market participation by smallholders (Akpo et al. 2020; Hillyer et al. 2006; Manda et al. 2020; David 2004). Périnelle et al. (2021) looked at the implementation of a more participatory approach to seed systems that could allow smallholders to be involved in the system outside of only cultivation. This could be important for promoting higher adoption rates and maintaining high seed demands which makes the legume seed business viable. Some of the papers (*n* = 4) suggested that specific training (for seed value chain actors and farmers) on newly introduced varieties or technologies increases adoption (and seed demand which is important in enhancing seed system performance) (Boadu et al. 2018; Mahama et al. 2020; Olatunde et al. 2021; Oyetunde-Usman et al. 2021). Two papers (*n* = 2) outlined improved storage methods to reduce losses (Baoua et al. 2012; Koona et al. 2007). This is important in ensuring that seed is not lost to insects and diseases—thereby ensuring seed access and availability.

**Table 6:** Options for scaling legume seed systems. (*x*) in the first column refers to the number of papers that mentioned this scaling option, crop is listed as “multiple” when the option applies to a range of legume crops.

Options	Crops	References
Increased support of new seed enterprises (training in production, marketing, etc.) (2)	Multiple	Akpo et al. (2020); David (2004)
Development and mainstreaming of seed storage technologies (1) to reduce post-harvest losses	Cowpea	Baoua et al. (2012)
Increased provision of information to smallholder farmers on the benefits of growing improved legume varieties (5) under intercropping or crop rotation systems	Chickpea, pigeonpea, common bean, cowpea	Haileyesus and Mekuriaw (2021); Gwenambira-Mwika et al. (2021); Nassary et al. (2020); Rusinamhodzi et al. (2012); Sauer et al. (2018)
Use of local knowledge (1) in developing farmer-preferred legume varieties can lead to increased seed demand	Multiple	Hillyer et al. (2006)
Location-specific planting of improved legume varieties (2). This is important in identifying the most adaptable and appropriate legume varieties for specific farming regions	Groundnut	Hoffmann et al. (2018); Nord et al. (2021)
Increased integration of formal and informal systems (1). Combining these two seed systems can have synergistic benefits on seed availability and access to remote smallholders	Multiple	Kilwinger et al. (2021)
Increased market participation by smallholders (1)	Cowpea	Manda et al. (2020)
Provision of specific training/ extension (1) to legume breeders, seed scientists, and farmers	Cowpea	Martey et al. (2021)
The use of participatory research methods (1) in developing new legume varieties can boost adoption and demand for new varieties	Multiple	Périnelle et al. (2021)
Strengthen community seedbanks (1) to ensure continuous access to underutilized legume varieties	Bambara nut	Sidibe et al. (2020)
Introduction of more flexible policy frameworks (1)	Multiple	Kuhlmann and Dey (2021)

For smallholders, food security is a main priority, and therefore, high yield cannot be sacrificed for other secondary traits (e.g., early flowering). The key challenges for strengthening legume seed systems revolve around demonstrating how legumes can benefit livelihoods when adopted by a farmer. Intercropping of legumes with cereals is one option to produce legumes, which was raised multiple times as a practice that can have positive impacts on yield and cropping systems (Gwenambira-Mwika et al. 2021; Rusinamhodzi et al. 2012; Haileyesus and Mekuriaw 2021). Another farming practice that is highlighted for legumes is the use of ridge tillage (Akinyemi et al. 2003). If post-harvest losses are affecting farmers, options for improved storage methods have been identified which can facilitate the adoption of legume seed in areas where it was previously limited (Baoua et al. 2012; Koona et al. 2007). Support for seed enterprises is another important option to improve seed access. Market participation has also been shown to be beneficial to both farmers buying and selling seeds as it can increase incomes and access to improved varieties (Akpo et al. 2020; Hillyer et al. 2006; Manda et al. 2020; David 2004). It has also been highlighted that the adoption of improved varieties increases when specific training is provided. Offering participatory approaches to training, using extension services and demonstrations along with local knowledge could increase trust in new varieties and improve levels of adoption (Boadu et al. 2018; Mahama et al. 2020; Olatunde et al. 2021; Oyetunde-Usman et al. 2021; Périnelle et al. 2021).

Another entry point to strengthening legume seed systems is creating an environment that promotes investments in the legume seed development space by private companies. To achieve this, we must look at the barriers to investments. On the breeding side, some challenges are inherent to the biology of legume crops (e.g., difficulty in crossing for some species due to their reproductive biology), while the seed multiplication ratio can act as an obstacle to increasing seed quantities for some legumes (e.g., groundnut 1:8, soybean 1:16, peas 1:19, cowpea 1:40). In addition, profit margins along the legume breeding and seed supply value chain (especially where the target market are smallholders) are not sufficient to sustain investment in legume breeding and seed systems. These factors act as a disincentive for private sector investment in legumes. Indeed, Rubyogo et al. (2016) highlight that the supply of new bean varieties has been generally left to NGOs, farmer organizations, and government bodies, with the private sector tending to focus on more profitable crops (e.g., maize, soybean, commodity crops). Furthermore, the provision of free or subsidized seed by NGOs and government bodies are two additional barriers that can discourage the commercial sector from investing time and capital in supplying seed directly to smallholder farmers (Tripp and Rohrbach 2001). However, market opportunities for seed companies can arise from input subsidy schemes run by government bodies and some NGOs. Supply of legume seeds to these organizations can provide an opportunity for sales of larger quantities of legume seeds and repeat sales.

Policy and regulatory environments were also a focus of some studies (*n* = 2) (Branca et al. 2021; Kuhlmann and  Dey 2021). Seed policies can act as either a barrier or an enabler for smallholder legume seed access (Poku et al. 2018; Okry et al. 2011)—depending on the nature and flexibility of regulations around varietal selection, varietal identity, varietal breeding, release, certification, and sale. Where a flexible regulatory approach is taken (e.g., the use of quality declared seed is accepted), this can create opportunities to increase seed access for smallholders (Branca et al. 2021; Kuhlmann and Dey 2021). In relation to seed system scaling, some similar barriers to adoption arise continuously throughout the studies in this review, with access to seed and improved varieties being the most significant. We also noted that smallholder households are usually positively impacted by the adoption of an improved legume variety, and therefore, it is important that seed systems are strengthened. In addition, options for the scaling of seed systems which are mentioned in the literature generally focus on making legume cultivation more lucrative and communicating to farmers the benefits of producing legumes, particularly improved varieties. Indeed, regulatory frameworks are required to better support community seed networks (Abebe and Alemu 2017). Some consider that seed laws favor the private sector which generates tensions and trade-offs with the informal seed system (Wattnem 2016). For effective legume system scaling, it is necessary to consider the seed laws for each country to investigate how they are enabling smallholders to access seeds of improved legume varieties.

## Conclusion

We systematically reviewed 129 research articles that focused on legume seed systems in SSA. Our results indicated that, although both formal and informal seed systems exist in SSA, there is a strong reliance on the informal system by smallholder farmers in sourcing seeds of non-cash crop legumes. The adoption of legume varieties, as identified in this study, was found to be influenced by various factors including seed costs, gender preferences, limited access to new variety information, inadequate and untimely availability of seeds in sufficient quantities from the formal seed system, inefficient demand estimation mechanisms within the formal seed supply chain, suboptimal seed quality within the informal system, and lack of high-yielding varieties that are tolerant to insect pests and diseases due to insufficient investments in legume breeding. In scaling legume seed systems, we identified several constraints including restrictive policy structures, limited investment in legume seed research, climatic vulnerabilities, and health shocks (such as the COVID-19 pandemic). Options identified to tackle some of these constraints included the provision of specific training (to breeders/seed scientists and farmers), incorporating farmer knowledge in seed development (to boost seed adoption rates), supporting local seed enterprises (technically and financially), and enacting more flexible policy instruments that support non-staple crop production. We consider that legume system scaling hinges upon the availability of an enabling policy environment and technical support structures. Our findings show that despite the increased interest in legumes, there are still some notable research gaps that require further investigation. These include a lack of research that explores the legume value chains and market dynamics in SSA. These two issues are important in improving the viability and profitability of legume seed production. There is also a need to assess the policy and institutional mechanisms that may need adjustments to create a more enabling environment for the existing legume seed systems in SSA. Lastly, future research must consider gender dynamics in seed systems—as access to legume seeds can be segregated by gender roles.

### Supplementary Information

Below is the link to the electronic supplementary material.

Supplementary file1 (DOCX 230 KB)

## Data Availability

The datasets (or search results) generated and analyzed in this review article are available from the corresponding author upon reasonable request.
