# Supplementary material for: Legume seed system performance in sub-Saharan Africa: barriers, opportunities, and scaling options. A review
Source: Agron Sustain Dev. 2024 Mar 26;44(2):20. doi: 10.1007/s13593-024-00956-6 (PMC10965649; doi:10.1007/s13593-024-00956-6)
Supplement: Supplementary file 1 — Supplementary file1 (DOCX 230 KB) [file 13593_2024_956_MOESM1_ESM.docx]

**Supplementary Materials**

**Supplementary Table 1. Number of results yielded by each search term and number of papers extracted from each database.**

|  | Search 1 | | Search 2 | | Search 3 | | Search 4 | |  |
| --- | --- | --- | --- | --- | --- | --- | --- | --- | --- |
| Database | Total Results | Papers extracted | Total Results | Papers extracted | Total Results | Papers extracted | Total Results | Papers extracted |  |
| Web of Science | 2,334 | 25 | 6,420 | 75 | 357 | 74 |  |  |  |
| Science Direct | 6,552 | 75 | 1,034 | 31 | 477 | 13 | 417 | 6 |  |
| PubMed Central (PMC) | 3,001 | 26 | 869 | 13 | 347 | 5 |  |  |  |
| ProQuest | 2,605 | 30 | 782 | 9 | 474 | 14 |  |  |  |
| Google Scholar | 1,020 | 5 | 44 | 2 | 135 | 2 |  |  |  |
| TOTAL | 15,512 | 161 | 9,149 | 130 | 1790 | 108 | 417 | 6 |  |
| TOTAL SEARCH RESULTS | 26,868 |  |  |  |  |  |  |  | 26,868 |
| TOTAL EXTRACTED | 405 |  |  |  |  |  |  |  | 405 |
| TOTAL (DUPLICATES REMOVED) |  |  |  |  |  |  |  |  | 319 |
| TOTAL USED IN REVIEW |  |  |  |  |  |  |  |  | 129 |

**Supplementary Table 2**. Overview of research papers on legume seed systems, including findings and recommendations

| Author, Location | Methodology | Objectives | Key findings | Recommendations |
| --- | --- | --- | --- | --- |
| (Mulesa, 2021), Ethiopia | Household surveys (n = 80), Focus group discussion, document review, key informant interviews with actors  Year(s) of study = 2017- 2018 | To compare seed systems in two districts of Central Ethiopia characterized by subsistence teff cultivation and commercial wheat production and relate this to the country’s pluralistic seed system development strategy (PSSDS) | Farmers in both districts used a range of seed sources. Seeds were primarily sourced from the informal system. Seed insecurity affected households in both districts, there were discrepancies between what the seed farmers said they preferred and the seeds they used. There was limited availability of improved varieties and certified seeds of improved varieties specifically, seed quality challenges from some sources. Access to preferred seeds and information is impacted by the sex, age, and wealth of the farmer. | Political, organizational, and economic interests within key institutions present major obstacles that must be overcome to develop the seed system in an integrative and inclusive manner. |
| (Otieno et al., 2021), Kenya, Tanzania, Uganda | Survey of rural farm households (n = 1001) across five study sites  Year of study = 2016 | To explore differences in seed networks accessed by women and men farmers for three major food security crops—beans, finger millet, and sorghum. | Women, on average, have fewer connections to experts and farmers’ groups than men but have better social networks with other farmers across various farming systems. Women’s and men’s networks are clustered by gender and men’s networks are more likely to exchange improved seed. Women’s networks, though sometimes larger, are less likely to exchange improved varieties. Women farmers may also depend more on farmer-to-farmer networks than men due to their relative isolation from other seed and information sources | There is a need for careful attention to the different implications of seed policies, market interventions, and other seed system reforms to support food security options that support men and women in SSA equitably. |
| (Akullo et al., 2018), Uganda | Literature search, interviews (n = 63), focus groups  Year of study = 2007 | Identify institutional processes in the creation of public-private partnerships (PPPs) for agricultural innovation.  Proposes to conceptualize technology as affordance, as opposed to viewing technology as an input. | Shows that institutions are predominantly understood as organizational arrangements. | Policies and projects that include institutional support should identify and develop their abilities to accommodate different groups and create a balance between different institutional styles in the implementation of development goals. Improving the capacities to support institutional change can be things such as creating meeting places, organizing events where people can voice their opinions, and finding ways to resolve conflicts. |
| (Almekinders et al., 2020), Ghana, Kenya, Malawi, Zimbabwe | Interviews (n = 270)  Year of study = 2010 | To better understand how introduced seeds diffuse among communities | 2–3 years after the trials had been organized, more than 90% of the farmers who were involved in the trial activities and who were given an input package with 1-5kg legume seed had shared this seed, with approx. four other farmers. Second-generation farmers (not directly involved in the scheme) shared their seed less frequently. 80% of all the seed sharings were of 1–2 kg of seed given as a gift. Only 5% of the sharings involved a cash purchase. Over half of the seed sharings were with family members and approx. third were among friends. Men and women shared a similar amount, and both shared with members of their own sex. Information about rhizobium as an input for soy was shared by more than one-third of farmers, mostly by farmers who had themselves participated in the demonstrations. Extrapolation of data suggests that in addition to the 250,000 farmers who participated directly in the N2Africa demonstration trials, another 1,400,000 farmers may have received the seed of a new legume crop or variety through seed sharing among communities | Knowledge of the seed-sharing mechanisms may offer opportunities to influence the diffusion of seeds. Providing farmers with somewhat larger amounts of seeds, and emphasizing the importance of sharing seeds and information with relatives and friends could be an important factor in achieving a greater number of farmers using improved seeds. |
| (Andersen et al., 2019), Uganda | Surveys (n = 27)  Year of study = 2014 | Proposes a general framework for analyzing the networks of observed and estimated seed trade data that translates into a broad range of seed systems. | We found that the starting position in the network was critical for the progress of an epidemic and final epidemic outcomes. The efficacy of node centrality measures was evaluated in villages in the network to attempt to limit disease spread. | This analysis framework can be used to provide recommendations for a wide variety of seed systems |
| (Ayenan et al., 2017b), Benin | In-depth survey (n = 18)  Year(s) of study = 2011-2015 | To identify stakeholders involved in soybean seed systems in Benin, to identify their roles and connections, and to analyze how successfully the seed systems are performing. | Soybean seed systems in Benin are composed of an NGO-led formal seed system and a dominant informal seed system. **Stakeholders involved in these systems include researchers, farmers, traders, NGOs, and extension agents**. From the perspective of stakeholders’ these systems are not satisfactory in the effective production and delivery of quality seed. | Priority should be given to the introduction of new varieties and participatory varietal selection should be implemented to raise farmers’ awareness about the benefits of using improved varieties and good quality seed. Training of farmers in seed production and managerial skills are options that have been suggested to strengthen the informal system and begin the integration of both formal and informal systems. The use of information and communication technology will be important in seed diffusion and to improve the flow of information throughout the seed system. |
| (Beyene, 2010), Ethiopia | Survey (n = 160)  Year(s) of study = 2000-2001 | To assess the seed production and distribution strategies among smallholder farmers in eastern Ethiopia which has been introduced by the NGO, the Hararghe Catholic Secretariat | The results of the study show the important role of informal networks and social capital in accessing production inputs, information, and knowledge. The informal seed supply system which begins with the NGO has a huge potential to increase access to improved varieties of various crops, which will contribute to an increase in the well-being of adopting smallholder households. The practice of seed exchange is uneconomical to seed producers. | This study suggests that the potential to improve seed access can be further exploited if there is an establishment of seed banks, investment in human capital, removal of underlying constraints, and creation of reliable seed markets. |
| (Branca et al., 2021), Malawi | Comprehensive Value Chain (VC) Map of cereal-legume production, a SWOT exercise, surveys (n = 340), policy analysis  Year of study = 2017 | To develop a greater understanding of the VC. Based on a policy analysis, our objectives are to identify the ways in which the value chain interacts with policy. | VC analysis has shown the need for an improved policy environment that recognizes the relevance of the role played by other VC actors (e.g., cooperatives, traders, agro-dealers, researchers) in promoting the development of the VC, particularly in a way that benefits smallholders. | Contradictions across policies show the need to harmonize and improve existing policies to promote the development of smallholder-friendly value chains through (i) A review of current legal frameworks in an effort to improve them and remove inadequacies (ii) increasing the budget allocation accompanied by proper accountability measures to ensure judicious use of the funds which target policies (iii) the provision appropriate, qualified staff to implement the policies; and (iv) promoting the policies among communities and making smallholders aware of how they apply to them in order to ensure adherence |
| (Croft et al., 2018), Kenya | This experiment was carried out in a randomized complete block design with three blocks and four replicates for each variety within each block (n = 24), Survey (n = 302)  Year(s) of study = 2015, 2013 | Assessed the potential for formal or informal seed systems to meet the demand for seeds of high-quality indigenous vegetable crops. | As the data have shown, there is a significant need to develop and promote certified ALV seed varieties. Improving formal seed quality to match the quality of seeds saved by farmers is a necessity, as is the provision of a consistently high-quality product. Improving access to, and distribution of, formal seeds is still a challenge, and a low price is the most important factor in encouraging ALV seed purchase.  There is little support for the hypothesis that market access positively impacts formal seed adoption, but farmer resources are important to consider | By evaluating determinants of farmers’ seed purchasing behavior, this study concludes that informal seed systems have greater potential to meet the needs of the farmers and should be strengthened.  This study suggests that policymakers should use context-specific data to guide decisions on seed policy. |
| (David, 2004), Uganda | Case study  Year(s) of study = 1994-1997 | Suggests seed production by FSEs as a method for meeting two goals: to sustainably distribute and promote improved and new crop varieties and to establish a regular source of good quality seed of either local or modern varieties. | While FSEs may provide a sustainable solution to the issue of inadequate seed supply, there is still a challenge in the implementation and scaling up of this approach in eastern and southern Africa. | Linkages need to be fostered between farmers, researchers, agro-enterprise specialists, NGOs, and the formal seed industry. Seed policy reforms must be implemented and more research systems that consider farmer preferences must be institutionalized. As the model proposed in this study suggests, FSEs must be developed within the context of an integrated seed supply system. This ranges from traditional seed production at the farm level to the formal and commercial seed industry, with each element having well-defined and connected roles |
| (de Boef et al., 2021), Ethiopia, Myanmar, Nigeria, Uganda | Mobile application and web surveys (n = 145), focus group discussions  Year of study = 2020 | Synthesizes the outcomes of rapid assessments of the seed system that were conducted between May and June 2020 in Ethiopia, Myanmar, Nigeria, and Uganda, and identify lessons learned, to inform stakeholders in other countries facing similar challenges (due to the COVID-19 pandemic), and provide insights and options to help address these challenges when and where they occur | The root cause of many disruptions in the supply of seed to farmers was reduced mobility which resulted in seed and connected industries operating at reduced capacity. The cost of doing business during these times may have increased the scarcity and price of inputs beyond what farmers can afford. Formal sales of quality seed were perceived to decline due to delays in distribution, less promotion, and fewer farmers. Delays in the development and release of new varieties are likely to result in several seasons where farmers do not benefit from investment in crop improvement. Social distancing prevents stakeholders from meeting to exchange goods, services, and information, but information technology, which is slowly becoming more widespread in the system helps with this issue.  The pandemic has highlighted an increased structural weakness in the seed system, for which reforms are greatly needed | Structural weaknesses were well-known to partners in the four countries where rapid assessments took place. Prior to the pandemic, reforms were needed. Fortunately, the pandemic may be the catalyst for reform. Looking post-crisis, it is critical to maintain momentum and take the opportunity to address critical issues in the seed system. |
| (Delêtre et al., 2011), Gabon | Surveys (n = 191)  Year of study = 2011 | Investigated the relationships between regional patterns of cassava genetic diversity in Gabon and local networks of seed exchange. | The key to understanding the dynamics of crop genetic diversity lies not only in the factors that influence connectivity between farmer communities but also in the rules that govern the transmission of lineage within communities. Drawing a parallel between clones of yam and the origin of clans among Kanak people in New Caledonia, the link with botany to the general understanding of social interactions and identity was highlighted. It is seen that cassava clones “hitchhike” along bloodlines. | This study demonstrates that anthropology offers powerful a tool to help understand the dynamics of crop genetic diversity |
| (Edson and Akyoo, 2021), Tanzania | Key informant Interviews, surveys (n = 140)  Year of study = 2019 | Assessed the quality uncertainties in maize and vegetable seed and its impacts on market exchange between farmers and seed sellers in Kilolo district, Iringa Tanzania | In many cases, maize and vegetable seed varieties did not give the farmers results that were defined by seed companies in the seed description. This information disconnect between seed companies and farmers presents a level of seed quality uncertainty. | Policies to prompt knowledge transfer on good agricultural practices must be implemented for sustainable crop intensification which can lead to industrialization. Participatory variety development must be emphasized to ensure that the traits of crop varieties supplied in the market appeal to farmers. Preservation of land races to conserve biodiversity for future crop improvement programs is extremely important. |
| (Jones, 2017), Mali, Niger, Burkina Faso | Interviews (n = 513), Group meetings, and maps created  Year(s) of study = 2010-2013 | Describes and analyses efforts to develop formal seed systems in Sahelian West Africa over the past decade and identifies impacts on farmers of the social institutions comprising the formal seed systems. | The results show that the social and spatial extents of the formal and informal seed systems are extended and integrated through social institutions that hold the values of both systems. The impacts of current market-oriented agricultural development projects are, therefore, more than in the past in part because the social institutions associated with them have a broader vision for productivity and economic efficiency. | Identifying and supporting social institutions, such as farmer organizations, can play a bridging role between formal and informal systems which is necessary to allow for agricultural development which meets the needs of many stakeholders |
| (Kansiime et al., 2021), Tanzania | Focus group discussions, key informant interviews (n = 142)  Year of study = 2019 | Assessed sustainability factors post-Good Seed Initiative project and explored the prospects for scaling out the approach to wider regions | Farmer seed production under both models continued to thrive, creating avenues for diversifying income sources and contributing greater than 50% to household incomes. Farmer seed production led, in part to increased availability of quality vegetable seed, especially in areas that are less served by the formal system. However, farmers producing QDS faced a lack of access to foundation seed, inspections, and seed testing services, which are important for quality seed production, and this was a challenge | Farmer-led seed systems, especially QDS, must be supported by the government to develop an appropriate seed system that meets the needs of smallholder farmers. Adoption of gender-inclusive approaches, particularly in contract farming is important to support female and male farmers equitably |
| (Kilwinger et al., 2021), Rwanda | Interviews (n = 390)  Year of study = 2019 | To understand seed sourcing practices of different farmers in order to inform the development of seed business models that serve all farmers | This study finds that commercial farmers have better access to formal seed sources than smallholders. However, most farmers in all typologies accessed new varieties and quality cassava seeds through informal channels. | Clarifications on the differences between farmers and their willingness to pay, the roles of seed degeneration, cost-benefit, and analysis are important requirements for the development of cassava seed business models. Tailoring seed systems and value chains can have a high potential as it acknowledges the needs of various farmer types, however careful coordination is needed to ensure that certain approaches do not conflict with or contradict others. |
| (Labeyrie et al., 2016), Kenya | Interviews (n = 197)  Year of study = 2015 | Investigated how farmers’ membership in three major social groups impacts sorghum seed exchange networks in a cultural contact zone on Mount Kenya | Seed exchanges and marriage ties are interrelated, and both are limited between the Mbeere and the other groups and are frequent between the Chuka and Tharaka groups. Patrilocality and ethnolinguistic endogamy, which are traditional in the groups studied, and they impact seed diffusion and are involved in crop genetic diversity flows. | These findings pave the way for improving crop metapopulation models for diversity studies through a more realistic understanding of gene flows through seed exchange, considering farmer relationships and social networks |
| (Madin, 2020), Ghana | Surveys (n = 429), interviews, focus groups  Year of study = 2019 | Looks at a case study of Northern Ghanaian Savannahs to examine politics and seed security among smallholders | These results offer an example of how smallholder vulnerabilities to seed insecurity are impacted by historical ethnic conflicts and political and neoliberal economic policies in ways that may not be considered in the current narratives of seed security | The key to achieving seed security is a better understanding of how local and national politics influence perceptions and affect access to high-quality seeds and seeds which farmers want. |
| (Madin et al., 2022), Ghana | Surveys (n = 429), interviews, focus groups  Year of study = 2019 | To assess smallholder experiences of seed insecurity and how household socioeconomic characteristics affect these experiences, understand how seed insecurity changes throughout the year and identify adaptation strategies to increase seed security | The findings show that major determinants of seed security in this region include village location, access to information, credit, and tractor-plowing services. The results also showed that seed security is often disrupted by factors other than climate change, including conflicts between farmers and herders, and the use of synthetic farming inputs. It was found that some of the identified determinants are also seeds in food security studies | This study argues that holistic efforts to resolve such factors are required which will help to achieve both seed and food security among smallholders |
| (Marimo et al., 2021), Kenya, Tanzania, Uganda | Survey (n = 1001)  Year of study = 2016 | Examines how seed systems enhance access to seeds, and information for adaptation to climate-related challenges in farming communities in Kenya, Tanzania, and Uganda, as well as how gender roles and institutional dynamics influence the process | Early-maturing, heat-tolerant, high-yielding, and pest- and disease-resistant varieties were all traits preferred by farmers. Seed systems of the crops studied are mostly informal—overall, 68% of women and 62% of men use their own saved seed. This shows female farmers' greater dependence on ‘informal’ sources of seed and information. Only 21% of respondents reported interacting with formal organizations. Both formal and informal organizations play important roles in providing improved seed/information which are both tools for climate adaptation | There is a need to support the further development of connections between informal and formal systems, building social networks already present. Inclusive and gender-responsive context- and country-specific seed interventions will ensure equitable outcomes, increase women’s empowerment, and strengthen both formal and informal seed systems for more effective climate-change adaptation |
| (McGuire and Sperling, 2016), Malawi, Kenya, DR Congo, Haiti, South Sudan, Zimbabwe | Interviews (n = 2592),  Year(s) of study = 2009-2012 | Documents the degree to which the informal system remains the seed source, particularly in Africa. | Farmers access 90.2 % of their seed from informal systems with 50.9 % of that coming from local markets. 55 % of seed is paid for by cash, indicating that smallholders are making financial investments in seed, even in informal systems. | The data show that seed system strategy has to focus more on smallholders and that precise goals have to drive the strategies used to move towards more impact-oriented seed system development. These results show that seed channels – formal, informal, and integrated are working to reach smallholders with seed products and information that they want and need |
| (McGuire and Sperling, 2008), Ethiopia | Surveys (n = 399)  Year(s) of study = 2005-2007 | Focuses on farmers’ use and assessment of crisis assistance within Ethiopia, where seed aid has been present for 34 years. | Farmers’ abilities to strategize and negotiate variability in seed availability are not being addressed by current approaches. | The most beneficial interventions will be those that enhance farmers’ choice and flexibility, such as timely provision of a diverse range of crops, or vouchers/cash to allow farmers to purchase seed or other items. Farmer seed systems are complex and generally resilient to stresses. Aid approaches need to consider the strength of local governance and institutions and need to work with farmers’ practices. |
| (Nordhagen and Pascual, 2013), Malawi | Household Survey (n = 11,280),  Year(s) of study = 2005-2006 | Addresses the relevance of agricultural seed systems in terms of the production and distribution of seed in the context of climate change adaptation. | Close to half of the households sampled nationwide used purchased seed and climate-related shocks significantly increased the use of purchased seed when all crops were viewed together. In the case of local OPV maize, the local market has few access barriers, with broad use and equal likelihood of use across farmers of all wealth and education levels. This contrasts with private seed sources which are used more by farmers with higher levels of wealth and education. This result is in line with other findings (Sperling et al., 2004) that market-sourced seed is important to seed security. Local markets have been shown resilient to shocks and are key following disaster periods, as they are lacking seed entirely. | In Malawi, the expansion of high-yielding maize provided increased food security. Additional highly productive varieties, produced through formal breeding programs with farmer participation will also be important in increasing food security. Such policy efforts in Malawi, however, will need to consider a need for greater varietal turnover under a changing climate. |
| (Nyantakyi-Frimpong, 2019), Ghana | Participatory Geographic Information Systems (PGIS), analyses of seed bank inventory, Interviews (n = 144)  Year(s) of study = 2013-2017 | Demonstrates how poor people excluded from the project of development challenge both its performance and its premise. | This study provides insights into the value of indigenous ecological knowledge. Furthermore, this study suggests that seed banks are impacted by gender dynamics at a village level. Indeed, the gendered micro-politics of resource access and control had major impacts on the results of the project. Further insights from the study focus on intersectional dimensions of difference. Recent research on FPE shows that by considering intersectional positions, and how these interact with each other, we can better understand power relations and how these affect access to resources. Additionally, this study considers how to make politics more visible in FPE, and indeed in political ecology more broadly. While significant research has been done on the politics of resource access and control, it is often difficult to show precisely how these politics occur in communities. | Seed bank projects could exist alongside efforts to support farmers in saving their own seeds. There is also a need to reconsider the narratives that community-level institutions are always more efficient and more trusted. This thought process does not consider the nuances of village politics and social relations. In order to prevent control by the local elite, village seed bank leaders should not hold these positions for more than two years. Additionally, even if women are not seen as leaders due to cultural norms, as was the case in this case study, they should still be involved in the governance of seed banks. |
| (Okry et al., 2011), Guinea | Focus group discussions, Surveys (n = 132)  Year(s) of study = 2007-2008 | Analyses the rice seed system in Guinea with the goals of assessing how organizational settings affect seed supply to smallholders and suggesting institutional changes that would favor seed service and adoption of varieties. | Results suggest that the current institutional settings and views of stakeholders from the formal seed system can limit smallholder farmers’ access to seed. Seed interventions over the last 20 years have depended on the national extension system, research institutes, NGOs, farmers’ associations, and contract seed producers to ensure seed delivery. Although local seed dealers play a central role in providing seed to farmers, governmental organizations operating in a linear model of formal seed system development have so far not filled this role. | This study suggests the participation of local seed dealers in seed development to create stronger connections between the formal and the informal seed systems and improve smallholder farmers’ access to high-quality seed from the formal system. |
| (Poku et al., 2018), Ghana | Process Net-Mapping, Interviews (n = 71)  Year(s) of study = 2015-2016 | Analyses the challenges faced by governance in seed systems. | The empirical evidence is in line with the theoretical considerations in the view that governance challenges affect all stages of the seed supply system. These challenges include limited involvement of smallholders in choosing breeding priorities, low levels of private sector participation in source seed production, limited capacities of the public regulatory body to ensure high seed quality through seed certification, and too much dependence on a weak public extension system to promote improved varieties. | These findings show that there is a need to pay more attention to the political economy of such seed system reforms. The findings indicate that there should be further emphasis on using the roles that the public sector, the private sector, and the underdeveloped third sector can play in ensuring that smallholders have greater access to improved seeds. |
| (Quarshie et al., 2021), Ghana | Key informant interviews (n = 15), Surveys (n = 110)  Year of study = 2018 | To increase understanding of how Early Generation Seeds value chain constraints limit the sale and adoption of High Yielding Varieties (HYV) and improved maize seed by smallholders in Ghana. | Seven main challenges were identified that limit the expansion of HYVs: (1) the capacity of public bodies is limited, (2) the capacity of the emerging private sector is constrained, (3) a lack of fair and appropriate contracts between stakeholders (4) land-tenure challenges, (5) forecasting of farmers’ demands for seeds by research institutions and seed producers are weak, (6) low levels of marketing of improved maize seeds, and (7) few institutions control the seed supply | Improving the maize seed value chain must be pursued through public and private sector relationships that acknowledge the important roles of various stakeholders within the value chain |
| (Rattunde et al., 2021), Mali | Case study  Year of study = 2The seed | The seed network is examined to determine how the network developed and what it does, varietal diversity, varietal performance, and organizational models present as a result, the elements of the traditional seed system that remained were improved or were changed. | The case demonstrates how creating a framework for collaboration, enabling actors and organizations to accept responsibility while maintaining decision-making at the local level, creates opportunities for transitioning agriculture and food systems towards increased sustainability and resilience. | Contributions to the transformation of agriculture and food systems towards sustainability and resilience can thus be made using this approach that emphasizes local livelihood and community goals and leadership in a network where global development agendas are the goal. |
| (Ricciardi, 2015), Ghana | Surveys (n = 91)  Year of study = 2013 | To identify farmers who can distribute open-pollinated seed through the social networks they exist within and evaluate if this method of distribution through social networks provides improved varieties to groups that alternatively would have limited access. | A number of concerns are seen in this analysis: i) control of resources in the hands of a single central farmer may lead to unequal distribution of seed as farmers use this control to maintain their position of power within the network ii) networks that appear similar may be difficult to compare due to a difference in scale. These findings show that the analysis of social networks is a useful tool in understanding the socioecological complexity of informal seed systems, however, this analysis must also consider the power structures in social networks when proposing to use them to disseminate resources. | Future studies should focus on when and how to inform smallholders on how they can access informal seed and steps communities can take to develop a more equal distribution of seed and other resources. Future research should identify how to use social network analysis to create collaborations between conservation groups and local stakeholders to develop inclusive conservation initiatives using local knowledge. |
| (Sperling et al., 2021b), Tanzania | Survey (n = 298)  Year of study = 2019 | Explores business within the informal seed system, focusing on the yellow bean in Tanzania. | Results showed that traders both buy and sell grain and informal seed. Informal seed is an important product, it comprises 15% (non-sowing period) to 40% (sowing period) of trader business. During the year studied, 100% of the yellow bean seed was sourced from the informal system, valued up to $US 4.35 million among those sampled. **Informal and formal systems have obvious linkages, as over 60% of the beans sampled are derived from improved varieties**. Informal traders are particularly important for sustaining the grain business, providing the core of the seed business, and moving varieties at scale. | Improved efforts are needed to link the informal system to formal research and development partners in order to achieve more widespread impacts |
| (Sperling et al., 2021c), Zambia, Zimbabwe, South Sudan, Malawi, Kenya, DR Congo, Madagascar, Ethiopia, Burundi | Seed system security assessments (SSSAs), Surveys (n = 2779)  Year(s) of study = 2009-2017 | To provide increased support to the channels smallholders mainly use and, especially, to increase access to improved varieties through dynamic business endeavors. | Findings show that approx. 50% legume seed is bought as opposed to saved, given as gifts, or obtained through a seed input scheme. **89% of purchases occur in the informal system, largely in local markets**. The use of agro-dealers to obtain legume seed is minimal. | Adjustments to formal system delivery can help extend reach but major improvements require strong innovation within the informal system. A recent review of seed legislation across several countries proposes that there may be opportunities for development of this kind. |
| (Sperling et al., 2020), Malawi, Kenya, DR Congo, South Sudan, Zimbabwe, Zambia, Madagascar, Ethiopia, Burundi, Tanzania | Surveys,  SSSA (n = 287)  Year(s) of study = 2009-2018 | To develop knowledge of local seed markets by focusing on the supply side, stakeholders who provide seed, particularly when seed is most in demand. | Traders have important roles to play in ensuring all aspects of seed security are addressed, especially in areas that have the least access to resources. One role traders hold is procurement and seeking out supplies to help with stress. Traders sell certified seed in small packs in areas where formal seed enterprises have narrow reach—however national seed laws limit this practice. Traders have engaged in efforts to improve seed quality by using improved storage methods such as hermetic bags, organizing varieties, and keeping warehouses at a high standard during times of relief intervention. | Traders should be involved when bodies are attempting to provide seed aid as they have strong connections to smallholder farmers. Donors should view traders as an important asset for strengthening the seed security and resilience of smallholders. |
| (van Niekerk and Wynberg, 2017), South Africa | Focus groups, (n = 43), key informant interviews (n = 15), Participatory mapping. Year(s) of study = 2011-2012 | Examines a case study from KwaZulu-Natal, South Africa which looks at traditional seed exchange systems and how they reinforce social relations and provide a safety net. | Findings suggest that traditional crops are the core crops grown by farmers; seed in these crops is valuable; and exchange among social networks is complex. Seed systems of traditional crops are an important element of food sovereignty and can fill gaps left in times of unexpected shocks. They are also important socially and culturally within families and communities. | Future research should further examine the complexities of seed exchange networks, particularly how they interact with and incorporate modern varieties in rural areas of South Africa, and this may impact food sovereignty and social cohesion. |
| (Kuhlmann, 2021), Colombia, India, Vietnam, Kenya, Peru, Brazil, Tanzania, Myanmar | Case studies, key expert consultations  Year of study = 2020 | Addresses knowledge gap surrounding the role of law and regulation in the creation of links between the informal and formal seed systems and creating a more inclusive seed system that is more effectively governed. | This study finds that more flexible regulatory approaches and practices are very important in developing and enhancing connections between formal and informal seed systems. | Any model for seed regulation will be context-specific, and differences in regulatory systems may indicate potential approaches for improving access, availability, and affordability of quality seed. National and local governments may have the option to adapt policy and regulatory options to local priorities. Flexibility can be integrated into other formal seed systems, reducing gaps between formal and informal seed systems. Local approaches can be highly resilient in the face of market disruptions, such as those caused by the COVID-19 pandemic. |
| (Mwangi et al., 2020), Kenya | Survey (n = 383)  Year of study = 2019 | Examines seed security among smallholder sweet potato producers in Kenya and how this seed security impacts household food security. | Results of this study show that our respondents experienced mild seed insecurity with a score of 4.8 (maximum score = 12). Results also show that seed security had a positive and major impact on food security. Seed access is shown to be the most critical element influencing food security. The regression results further show that wealth index, distance to the market, income, and education level had a positive impact on household food security. | These findings put emphasis on the importance of seed security for orphaned crops, especially among low-income households as an approach to increase resilience and improve household food security thus improving food and nutrition security nationwide. The modified Seed Security Experience Scale (SSES) employed in this study is shown to be robust enough in assessing seed security for a particular crop and region and it is proposed that it could be used in other crops and regions. |

**Supplementary Table 3. Research studies focusing on specific legume varieties in SSA.**

| Author, Location | Methodology | Objectives | Key findings | Recommendations |
| --- | --- | --- | --- | --- |
| (Banla et al., 2018), Togo | Interviews (n = 180)  Year of study = 2018 | To identify constraints to groundnut production and assess farmers’ preferred traits. | Traits pertaining to yield such as pod yield and pod size were the most important. Pests and diseases were the greatest constraints to groundnut production. Among diseases, farmers in all three regions indicated that late leaf spots are of economic importance. No gender differences were observed for the perception of constraints and groundnut trait preferences. Age and gender had significant influences on land size. Besides, farmers have pointed to the lack of improved varieties and the lack of seeds highlighting the necessity of a sustainable groundnut seed system that has links with a strong breeding program. | There are opportunities for plant breeders in Togo to develop groundnut cultivars that will meet farmers’ preferences and increase the adoption rate of improved varieties. The development of strong seed production systems can address the lack of availability of improved groundnut seed. A breeding program that permits interaction between a range of actors including farmers, traders, and seed companies increases the likelihood of adoption of new groundnut varieties. Groundnut breeding programs should consider that farmers perceive diseases as the major constraints to production. High-yielding groundnut varieties with large pod sizes and resistance to late leaf spots are likely to be adopted by groundnut farmers in Togo. |
| (Belayneh and Chondie, 2022), Ethiopia | A randomized complete block design with four replicates, (n = 22)  Year(s) of study = 2019-2020 | To evaluate the performance of groundnut varieties through farmers' participation and assessing their preferences | Among the tested varieties, *BaHajidu* (1805.84 kg /ha) was identified as the best-yielding groundnut variety, followed by *Bulki-01* (1805.50 kg/ ha) and *Werer-963* (1780.0 kg/ ha), respectively, while *Werer-962* variety has a lower yield (1536.30 kg /ha). The highest-yielding varieties *Bulki-01*, BaHajidu, and *Werer- 963* were preferred by farmers. | Based on farmers’ preferences and yields, these three groundnut varieties were recommended to be used in demonstrations and large-scale production in areas with similar agroecologies to the study zone. |
| (Giami, 2005), Nigeria | Samples boiled, steamed, or given no heat treatment (control). Protein quality was evaluated using a rat bioassay. (n = 4)  Year of study = 2003 | To determine the properties of raw, boiled, and steamed samples of selected advanced breeding lines of cowpeas. | Boiling was shown to be more effective than steaming in reducing the levels of antinutrients and improving the protein quality of the seeds. IT8ID-699 and TVx3236 lines were found to be nutritionally superior to IT82E-18 and IT84S-2246-4. | The cowpea lines studied here could possibly be combined into a single cowpea line and further improved by breeders to have other desirable cooking qualities, such as reduced cooking times and higher levels of water absorption during soaking. |
| (Ayenan et al., 2017b), Benin | Interviews, group discussion, (n = 302)  Year of study = 2015 | To analyze farmers’ practices and constraints related to pigeonpea production as well as identify farmers’ preferred traits in pigeonpea. | Pigeon pea is mostly grown by men. Approximately 98% of the pigeonpea growers associated pigeonpea with other crops, and the remaining 2% grew the crop on its own. Pigeon pea-maize (48.7%) was the most common cropping system. The type of crops associated with pigeonpea depended on the growing area (P < 0.001). Lack of improved varieties, low productivity, and lack of quality seed were major constraints to pigeonpea production. The pigeonpea seed system was essentially informal with self-saved seed (79%), purchase from fellow farmers or from local markets (12%), and gift/exchange (9%) serving as the seed sources. High-yielding, early maturing and resistance to pod borers were the main reported traits preferred by farmers. | Farmers should be supported in terms of access to quality seeds through extension services in seed production. Pigeon pea growers seek traits such as early maturing, high yielding, insect resistance, and large seeded varieties. Breeding and introducing varieties having those traits would increase the rate of adoption and subsequently, the productivity of the crop if there is also an adequate seed production and delivery system. In addition to improved varieties, decision-makers should create an environment for farmers to have access to inputs (seeds, fertilizers, and insecticides), credit, and markets. |
| (Merga, 2020), Ethiopia | Randomized complete block design (RCBD) in factorial arrangement with three replications.  Year(s) of study = 2017-2018 | To identify the best-performed common bean variety and determine the optimum row spacing for the common bean varieties tested. | The hundred seed weight and number of seeds per pod varied between the varieties and the different row spacing. Hundred seed weight was increased with row spacing size. Pod number per plant, nodule number per plant, grain yield, and harvest index were significantly affected by row spacing, variety, and the interactions between these two factors. The highest yield for variety-Nasir and variety-Goberesha were observed at 40 cm row spacing and the highest yield for the local variety was recorded at 50 cm. | Multi-locational experiments in more than one season could give valuable further information on the yields of these varieties. |
| (Misiko et al., 2008), Kenya | Plots were managed according to farmers’ practices and evaluated using participatory monitoring and evaluation approaches.  Year of study = 2004 | To understand the process of selecting soybean varieties by smallholders for soil fertility management in western Kenya. | Seven promiscuous varieties had better yields than local varieties. Farmers’ selection criteria fell into three categories relating to yield, appearance, and labor requirements. This study shows that farmer criteria for selecting varieties have similarities with scientific methods of varietal selection. | The study proposed that research activities involving farmers strengthen farmer experimentation skills, their understanding of N addition, and the role of P in soil fertility. |
| (Mligo and Craufurd, 2005), Tanzania | Ten cultivars were planted at six locations, and measurements were taken.  Year(s) of study = 1994-1995 | To measure biomass (BY) and seed (SY) yield of a set of phenologically diverse pigeonpea varieties to determine their adaptation to various environments in Tanzania. | The highest-yielding environment was at *Selian*, where mean temperatures were favorable. The best-adapted cultivars were ICP 7035, ICPL 90094, *Kat 50,* and QP37, which were all medium flowering (c. 150 days) types. Extra-early cultivars such as ICPL 86005 also showed potential, particularly for short-season environments. | This type of simple but robust analysis would be appropriate in many less developed countries, where the analysis and collection of more in-depth physiological data sets are often difficult and may help in identifying better-adapted genotypes of other crops.  It would also be useful to analyze longer-term weather data to establish a target mean crop duration, as well as to identify target environments. |
| (Mubaiwa et al., 2018), Zimbabwe | Interviews (n = 231), Focus group discussions, Practical observations, demonstrations  Year of study = 2014 | To gather knowledge on the production and utilization of Bambara groundnut, to identify its role in providing food and nutrition security sustainably for rural populations. | Results revealed a variety of Bambara nut processing techniques were used, such as boiling, soaking, roasting, and milling. Constraints to processing and consumption included long cooking time, difficulties with milling, and high firewood and water requirements. 50-80% of respondents in all districts consumed Bambara groundnut 1 or 2 times per week from August to December. **Preferred consumer attributes were taste, satiating effect, and nutritional benefits.** | The Bambara nut value chain lacks a grading system, which shows how relegated the crop is. The development of a standard grading system is highly recommended. The government is recommended to develop policy incentives for legume production. Knowledge sharing, education, building from existing strategies, and applied research are essential interventions for improving food and nutrition security through Bambara groundnut. |
| (Mwalongo et al., 2020), Tanzania | Interviews (n = 300)  Year of study = 2019 | Analyses factors underpinning the adoption of improved groundnut varieties among farmers to identify approaches for upscaling quality seed used for increased production in farming communities. | The empirical results showed that *Johari 1985, Pendo 1998, Naliendele 2009, Mnanje 2009, Mangaka 2009* and *Nachi* 2015, are the main six improved groundnut varieties used by farmers, with *Pendo 1998* having the highest adoption rate (17.1%). Furthermore, among the adopted improved varieties, *Nachi 2015*, is observed to be the most consistent high-yielding variety, ranging from 1100 kg/ha to 1500 kg/ha in all agroecological zones. | An integrated seed system development approach would sustainably enhance access to quality seed of new varieties in multiple areas. The promotion of new varieties along with complementary agronomic practices would incentivize farmers to adopt the new variety. Second, research, extension, and development organizations could make a difference by participating in the promotion of the new groundnut varieties to all stakeholders. This will allow farmers to easily identify quality seed sources. Third, the deployment of labor-saving machinery would enable old farmers to easily manage the labor intensity to grow groundnuts and benefit from the positive impacts linked to improved varieties. Fourth, the enactment of good policies within the seed system, such as comprehensive seed subsidies, will attract more seed companies to invest in groundnut seed production. |
| (Ngalamu et al., 2020), South Sudan | Randomized complete block design with three replications.  Year(s) of study = 2014-2015 | To assess the yield stability performance of improved cowpea genotypes across six environments in South Sudan. | IT90K-277-2 was the highest yielding while ACC004 was the lowest yield genotype. *Palotaka* was a highly discriminating and repeatable environment compared to the other testing sites. IT07K-211-1-8 and *Mading Bor II* were the most responsive genotypes, while IT90K-277-2 was the most stable and high-yielding genotype across all test environments. IT90K-277-2 can be grown by farmers across the region. | The study revealed that farmers in high-rainfall lowland areas should avoid growing improved cowpeas during the first season due to high insect and disease pressure. Farmers in lowland areas in SSA can tap into the stable performance of the best three genotypes for production and marketing to improve food security and household income. |
| (Nord et al., 2020), Tanzania | A modified split plot with three blocks replicated at each site.  Year(s) of study = 2016-2017 | To identify lablab accessions that are high yielding and stable as well as those that perform best when sole cropped or intercropped with maize. | Average grain yield was substantially reduced in environments with maximum temperatures greater than 33 ̊C, but biomass production yielded comparable amounts across high temperatures and in dry (<500mm rainfall) environments. There were trade-offs between biomass and grain yield across high-yielding cultivars. In a comparison of production and N fixation measurements, varieties were identified that may have high performance in both. | A recommendation from this study is that lablab production and accession evaluations be conducted using intercrop rather than sole conditions, as this is more applicable to small-scale farming systems. |
| (Ruelle et al., 2019), Ethiopia | Interviews (n = 1296)  Year(s) of study = 2016-2017 | To assess the impacts of multiple factors on legume species diversity, to measure the varietal diversity of five legume species, and to evaluate the conservation of legume varieties by farmer households and communities. | Legume species richness increased with altitude, relative household wealth, and area planted to legumes. The highest number of varieties were found for the common bean, followed by field pea, faba bean, groundnut, and fenugreek. The average number of varieties planted per household was ranged from 1 to 2 and often much lower than the number reported in the same community, which ranged from 2 to 18. Most varieties were rare, planted by less than 1/3 of farmers. Farmers named varieties planted by others in the same community correctly, which showed awareness of legume diversity at the community level. | Given that the ability to plant multiple legume varieties is limited by land size, policies need to strengthen community-level conservation. |
| (Deu et al., 2014), Mali | Surveys (n = 22), Randomized complete block trial with four replications.  Year of study = 2009 | Analyses the efficacy of farmers’ strategies for preserving varietal seed purity and genetic integrity of an improved line of sorghum. | Farmers were generally able to maintain the phenotype, as well as sustain and even improve the yield performance of their *Soumba* variety while at the same time genetically enriching their seed stock. | Training farmers on the effects of recycling and seed management practices, especially for farmers with key roles in the traditional system of seed exchange, could help to increase local seed system security and enable farmers with limited access to the formal system to access improved varieties of seed. |
| (Hoffmann et al., 2018), South Africa | Tested crop model against field trial data and performed crop model simulation runs for all cultivated land in Limpopo.  Year(s) of study = 2013/14, 2014/15, 2015/16 | To explore the effect of the cultivars and planting dates on groundnut yield. | Longer growth duration by cultivar ICGV 03796 appeared to be more productive than an earlier maturing cultivar added into the simulation analysis as a third cultivar. However, for the generally drier regions of Limpopo, this cultivar with shorter growth duration would indeed be more productive in *El Niño* years. | This study demonstrated that management recommendations the at provincial scale explicitly need to consider in more detail location-specific and season-specific approaches. |
| (Misiko, 2013), Kenya | Survey (n = 300), Key informant interviews (n = 15), Participatory learning trials (n = 6), Focus group discussions (n = 6)  Year of study = 2008 | Illustrates obstacles involved in Participatory Varietal Selection (PVS). | Findings reveal significant difficulties in PVS that resulted from unexpected factors such as new crop disease and floods. As a result, this majority engaged in impulse buying, due to a poor grasp of productivity factors underlying traits of the new germplasm. | PVS must be anchored within an integrated knowledge exchange system that addresses challenges within the interaction of smallholder social, spatial, and temporal contexts. |
| (Nchanji et al., 2021b), Kenya | Demonstrations, Participatory varietal selection (n = 93)  Year of study = 2019 | To understand similarities and differences between men’s and women’s varietal and trait preferences for biofortified and local bean varieties (landraces). | Results indicate that varietal and trait preferences between men and women farmers were slightly different. Women farmers have preferred landraces compared to men due to their availability, affordability, and accessibility. **High-yielding was the most prioritized trait by both men and women**. The findings support the long-held assumption that men prefer market-oriented traits and that women have a greater range of concerns than just the market. Characteristics such as education, age, marital status, and land ownership impacted trait preferences. | This study calls for the involvement of both men and women at the design stage of any breeding system to ensure both men and women farmers have access to varieties they prefer for food and the generation of income. |
| (Shiferaw et al., 2008a), Tanzania | Surveys (n = 240)  Year of study = 2004 | Evaluated the adoption and impact of two farmer and market-preferred and disease-resistant pigeon pea varieties that were developed and promoted in semi-arid Tanzania. | The data from northern Tanzania shows that **several households were constrained from adopting new varieties due to limited seed access**. Some potentially adopting farmers were from growing new varieties due to lack of access to seed. Yield losses due to disease decrease from about 50% for local varieties to just about 5% for improved varieties. | There is an urgent need to strengthen rural institutions and involve them in addressing market failures in both seed and output markets in areas with limited market infrastructure.  Promotion of improved varieties, combined with better local availability of improved seeds at reasonable prices offers the most promising policy mix to scale adoption. The participation of farmer organizations and the private sector can be expanded through better promotion, seed production, distribution, and marketing strategies. |
| (Agyeman et al., 2021), Ghana | Interviews (n = 113)  Year of study = 2019 | Analysed perceptions, constraints, and preferences for improved Bambara nut varieties among smallholder farmers in Ghana. | Farmers reported that Bambara nut is disease tolerant, that it can grow on low-quality soils, and can grow with low levels of fertilizer. They also reported that Bambara nut fixes nitrogen and is high in essential nutrients. **Farmer-preferred traits in an improved Bambara nut variety were ease of harvest mechanically, drought, disease, and waterlogging tolerance, nitrogen-fixing ability, high yielding, early maturing, and ease of cooking.** | Farmer-preferred traits should be considered within the breeding programs of improved Bambara nut varieties to achieve higher levels of adoption and thus have positive impacts on food security in these households. |
| (Mutari et al., 2021), Zimbabwe | Surveys (n = 176), focus group discussions, transect walks  Year of study = 2019 | To determine constraints to production and trait and varietal preferences of common bean and identify approaches for overcoming these barriers to production. | Approaches farmers employed to tackle these issues included mulching, ridges, reduced acreage, and cultivating in a way that retains greater levels of soil moisture. Farmers also irrigated crops at night and changed planting dates in order to address the issue of heat stress. **Heat and drought stress, early maturing and disease resistance were the traits most preferred by farmers.** | This study finds that breeders should use participatory breeding approaches in order to improve varieties and to develop new varieties which could be considered ‘climate-smart’. Varieties with traits such as early maturing and heat and drought stress will have a high potential for adoption in dry, hot areas. Farmers should receive training in contract farming. Additionally, the establishment of community seed banks could improve seed access, particularly during times when seed delivery or varieties fail, seed banks may also assist in preserving locally adapted varieties. The issue of power cuts to irrigation schemes can be managed using solar energy. |

**Supplementary Table 4. Research papers focused on the adoption of improved legume varieties.**

| Author, Location | Methodology | Objectives | Key findings | Recommendations |
| --- | --- | --- | --- | --- |
| (Agyeman et al., 2021), Ghana | Interviews (n = 113)  Year of study = 2019 | Analysed perceptions, constraints, and preferences for improved Bambara nut varieties among smallholder farmers in Ghana. | Farmers reported that Bambara nut is disease tolerant, that it can grow on low-quality soils, and can grow with low levels of fertilizer. **The greatest constraints to Bambara nut production are lack of capital and access to seeds of improved** varieties. | Farmer-preferred traits should be considered within the breeding programs of improved Bambara nut varieties to achieve higher levels of adoption and thus have positive impacts on food security in these households. |
| (Ahmed et al., 2016), Ethiopia | Structured questionnaire (n = 301)  Year of study = 2016 | Assessed how the adoption of improved groundnut varieties affected the well-being of the smallholders in Eastern Ethiopia. | Households assessed in this study experience a positive and significant impact on welfare when an improved groundnut variety is adopted. | Various factors that impact seed access should be addressed in order to increase adoption levels of improved groundnut varieties, which have positive impacts on the welfare of groundnut-producing households. |
| (Asfaw et al., 2012), Tanzania, Ethiopia | Surveys (n = 1313)  Year of study = 2008 | To evaluate how the adoption of improved legume varieties affects the welfare of rural smallholders. This is done by measuring household consumption and expenditure. | The adoption of improved agricultural technologies has a significant positive on both consumption and expenditure. Greater levels of consumption and expenditure as a result of increased income from improved technologies lead to lower poverty, and higher food security and both of these factors combined result in a more resilient household. Local access to seed and access to information about the new varieties are major constraints on the adoption of both pigeonpea and chickpea crops. | Policy is required to improve public extension services and institutions in rural communities. Promotion of improved varieties and increased access to improved seeds at affordable prices would be the most likely pathways to increase adoption. Small seed companies should be linked with agri-business development groups and cooperatives and agro-dealers should be utilized in order to distribute seed more effectively when produced. This assistance should be enabled through policy. By offering extension services training farmers in quality seed multiplication and production not only can greater amounts of improved seed be produced and access to improved seed can be given to more smallholders but these producers can also supply seed to the formal system. |
| (David et al., 2002), Tanzania | Survey (n = 300)  Year of study = 1998 | To examine how the availability of seed impacts the adoption of new crop varieties. | According to the results of this study low adoption of the *Lyamungu 90* variety was a result of limited access to seed along with minimal promotion of the improved variety, and unpredictable market demand which was partly a result of the inconsistent distribution of seed. Results of this study show that seed availability may be influencing adoption more than the lack of interest or preference from farmers. | In order to allow new varieties to become established in seed networks input schemes must supply seed to poor farmers for a number of consecutive years in a row. |
| (De Brauw et al., 2018), Uganda, Mozambique | Surveys (n = 1613)  Year(s) of study = 2006, 2007, 2009 | Examines how this project impacted adoption and awareness of nutrition. Additionally, the study looks at impact pathways that lead to adoption and looks at vitamin A intakes within households. | The project increased education among those involved on several important pieces of nutrition information. It was found however that education on these nutrition messages may not affect levels of adoption. If vitamin A intakes increased this was more often due to increased adoption of certain crops and not due to the education given to participants. Results show that the introduction of new crops can be successful on the condition that the new crop has the same or higher yields than the previous one and that the new crop meets consumer preferences. | This study suggests that costs could be reduced and impact could be increased if farmers were actively encouraged to share seed with farmers not directly involved in the project. Future studies will consider mechanisms for encouraging farmers to share planting material with their social network to extend the reach of projects such as this. |
| (Dessalegn et al., 2022b), Ethiopia | Surveys (n = 360), focus groups, key informant interviews  Year of study = 2017 | To find drivers of adoption of improved legume cultivars using a case study of chickpea and lentil production in Ethiopia. | Constraints to adoption found in this study are unequal access to seed, inputs, credit, and information. Women have less access to all these resources than men and the cost of production for female farmers is high. Land ownership is a main constraint for young farmers. Farmers responded to the challenges by saving seeds and mono-cropping lentils, despite declining soil and crop productivity, and increased occurrence of pests and diseases. In the case of chickpeas, it was found that cultural norms also discouraged the adoption of the improved variety. | Following this study, there is a need to address the challenges of inequality between male and female farmers and develop a seed system that meets the needs of all farmers regardless of gender or age. Such a system would (i) deliver research outputs both in the form of improved seeds and agronomic practices which are introduced through gender-aware extension services and (ii) develop a seed supply system that supplies standardized seed when, where, and to whom it is needed (iii) provides affordable credit to all types of farmers (iv) assist with the involvement of the private sector in with strict monitoring of the chemical inputs being supplied in order to protect consumers from price extortion. |
| (Dionco-Adetayo et al., 2002), Nigeria | Interviews (n = 195)  Year of study = 1999 | To analyze the factors that influence the awareness, training, and adoption of household soybean consumption. | The main factors influencing soybean consumption negatively were found to be a lack of awareness of the benefits of soybeans and insufficient training. Consumption of soybeans was encouraged by lower costs, better training, and health issues within the household. | The importance of awareness and education is emphasized in this study. Future projects hoping to implement greater use of a certain food item must consider the socioeconomic status of the household when delivering training and must make it context-specific to have greater impacts. Processing techniques that are easy make adoption more likely, and emphasis should be placed on the cost and health benefits of soybeans. |
| (Hambloch et al., 2021), Kenya, Uganda, Tanzania | Case study, Key informant interviews (n = 36)  Year of study = 2021 | To analyze three hypotheses in relation to sorghum seed systems in the 3 countries studied. | This study shows that the adoption of improved crop varieties and the role the private sector has in the development of rural communities is significantly impacted by agro-ecological, social, and political-economic contexts. | This study recommends that future projects should develop diverse seed delivery systems with links to both formal and informal systems. They should also examine the trade-offs involved in adopting seed technologies from a livelihoods perspective and consider that seed systems improvements are one option of many for the development of rural communities. |
| (Konja et al., 2019), Ghana | Surveys (n = 250)  Year of study = 2018 | To compare certified groundnut seed (CGS) and conventional groundnut (CG) production in terms of their profits. | The results of this study show that production using CGS gives greater profits and greater profit efficiency than CG production. Age, education, extension access, and meetings with farmer organizations all impact the profit efficiency of CGS, however, level of education, access to extension, and mobile phone access influence the profit efficiency of CG production. | Producers of CGS should increase the scale of their production in order to meet the demand for CGS in Ghana. This will require increased capacity for these producers. Additionally, farmers currently using CG production methods should receive extension services in production using CGS. |
| (Larochelle and Alwang, 2022), Rwanda | Survey (n = 1440)  Year of study = 2011 | Investigate the effects of the adoption of an improved bean variety on dietary diversity, food security, and food group consumption within smallholder households. | Dietary diversity was found to improve following the adoption of the improved variety, along with food insecurity being reduced. This is likely due to the increased income as a result of the use of high-yielding varieties. Higher household dietary diversity is linked to higher consumption of cereals, fish, seafood, and fats. | This study suggests that including impact evaluation in the introduction of a new technology allows for better recording of the impacts of the study. DNA analysis could be incorporated into impact assessments in order to correctly identify which varieties gave which results. This may be useful because often there are many varieties with common local names which can be difficult to differentiate. |
| (Manda et al., 2019), Nigeria | Surveys (n = 1,525)  Year of study = 2017 | Examines how the adoption of improved cowpea varieties can reduce income and asset poverty. | This study demonstrated that the adoption of improved varieties greatly improved per capita household income and asset ownership. Poverty and asset poverty were both found to be reduced by improved variety adoption. | Results suggest that cowpea seed systems require significant investments in order to improve seed availability for smallholders at prices they can afford. |
| (Mhango et al., 2013), Malawi | Surveys (n = 88)  Year of study = 2007 | Examines barriers and options influencing the adoption of legumes by smallholder farmers in the south of Africa. | It was found that smallholders grew legumes for a range of reasons outside of the benefits for soil cover and fertility such as infant nutrition, pest control, and vegetable and grain production for sale and for household consumption. Legumes producing edible grain were preferred by farmers and legumes were not sown in large areas. | Policies and education that help farmers access quality seeds and fertilizers for poor-quality soils are valuable. Legumes which improve soil quality through leafy residues and root systems, and which produce edible grain are beneficial for farmers in increasing food security, income, and soil health. |
| (Muoni et al., 2019), DR Congo, Kenya | Survey (n = 268)  Year(s) of study = 2016-2017 | Identify perceptions held by farmers regarding legumes and identify why farmers carry out certain legume production practices. | Of the farmers involved in this study most had knowledge of legumes and their traits, however much lacked knowledge on some important characteristics of legumes such as the control of soil erosion and the benefits to soil fertility. Information about legumes was most often accessed from radio and fellow farmers. Farmers with large numbers of livestock prioritized livestock feed as a function of legumes. Results found that farmers value short-term benefits of legumes such as food and income over long-term benefits, such as impacting soil quality. | Education and increased market access to obtain inputs and sell products will help farmers access more of the advantages of producing legumes. |
| (Mutari et al., 2021), Zimbabwe | Surveys (n = 176), focus group discussions, transect walks  Year of study = 2019 | To determine constraints to production and trait and varietal preferences of common bean and identify approaches for overcoming these barriers to production. | The major barriers limiting the production of common beans were found to be drought and heat stresses, power outages, poor soil fertility, and pod shattering. The sale of crops faced the challenges of high inflation, delayed payment, and contract breaches. | This study finds that breeders should use participatory breeding approaches in order to improve varieties and to develop new varieties which could be considered ‘climate-smart’. Varieties with traits such as early maturing and heat and drought stress will have a high potential for adoption in dry, hot areas. Farmers should receive training in contract farming. Additionally, the establishment of community seed banks could improve seed access, particularly during times when seed delivery or varieties fail, seed banks may also assist in preserving locally adapted varieties. The issue of power cuts to irrigation schemes can be managed using solar energy. |
| (Mwalongo et al., 2020), Tanzania | Interviews (n = 300)  Year of study = 2019 | Analyses factors underpinning the adoption of improved groundnut varieties among farmers to identify approaches for upscaling quality seed used for increased production in farming communities. | Age and gender, farmer group membership, availability of improved seed, and seed cost all affect a farmer’s decision to adopt an improved variety. Overall, male farmers are more likely to adopt improved varieties of groundnut than female farmers in this study. | An integrated seed system development approach would sustainably enhance access to quality seed of new varieties in multiple areas. The promotion of new varieties along with complementary agronomic practices would incentivize farmers to adopt the new variety. Second, research, extension, and development organizations could make a difference by participating in the promotion of the new groundnut varieties to all stakeholders. This will allow farmers to easily identify quality seed sources. Third, the deployment of labor-saving machinery would enable old farmers to easily manage the labor intensity to grow groundnuts and benefit from the positive impacts linked to improved varieties. Fourth, the enactment of good policies within the seed system, such as comprehensive seed subsidies, will attract more seed companies to invest in groundnut seed production. |
| (Nateebwa et al., 2017), Uganda | Survey (n = 249)  Year of study = 2016 | To investigate public sector Community-Based Seed Multiplication (CBSM) and its effectivity and identify factors impacting farmers' access to improved bean varieties. | The results of this study showed that the effect of public sector CBSM was significantly positive. Farmers to took part in the project had increased knowledge and increased access to bean seeds at the completion of the study. Access to seed was increased due to the higher income they earned from bean sales and due to the distance from their farm to the nearest bean market. CBSM membership benefitted farmers in rural areas more than those who were located close to formal seed markets. | This study recommends that public sector CBSM projects that are implemented in the future should particularly target farmers in rural and remote areas who have limited access to commercial seed providers. |
| (Nchanji et al., 2021b), Kenya | Demonstrations, Participatory varietal selection (n = 93)  Year of study = 2019 | To understand similarities and differences between men’s and women’s varietal and trait preferences for biofortified and local bean varieties (landraces). | **High-yielding was the most prioritized trait by both men and women**. Characteristics such as education, age, marital status, and land ownership impacted trait preferences. Despite having attractive traits, the Nyota variety increased labor, leaving women with less time for other tasks, and required additional inputs and therefore had some adoption trade-offs. | This study calls for the involvement of both men and women at the design stage of any breeding system to ensure both men and women farmers have access to varieties they prefer for food and the generation of income. |
| (Olatunde et al., 2021), Nigeria | Survey (n = 108)  Year of study = 2020 | Determined the adoption rate of high-yielding varieties of soybean and of inputs such as fertilizers, pesticides, and herbicides among soybean farmers. | The research found that the adoption of herbicides and pesticides is widespread while the adoption of fertilizers remains low for soybean farmers. Factors such as gender, age, household size, extension, and credit access have significant influence on the adoption of these technologies. Extension services have a positive impact on fertilizer adoption and the level of education and access to credit impacts the adoption of herbicides and pesticides. Female farmers are more likely to adopt herbicides but less likely to adopt fertilizers than male farmers. | This study recommends that the rural financial market should be strengthened to improve access to credit for farmers. Additionally, extension services that offer specific training in relation to fertilizer used in soybean should be offered in order to improve soybean productivity and increase livelihood benefits to farmers. |
| (Ronner et al., 2018a), Uganda | Demonstration trials, adaptation trials (n = 374)  Year(s) of study = 2014- 2015 | To examine changes in climbing bean production and the reasons for these changes. The farmers studied were from various geographical areas and socioeconomic backgrounds. | 70% of the farmers involved in the study continued the production of climbing beans following the end of the study. Factors influencing farmers to stop climbing bean production were poor weather, lack of seed, and lack of stakes. Performance of the trials was negatively impacted by late planting however this represents logistical difficulties with large-scale projects. Prediction of the use of various practices proved to be difficult due to differences within districts and household characteristics. | This study demonstrates the need for intervention projects to offer a selection of practices from which farmers can choose, in order for the intervention to be helpful in many contexts. |
| (Shiferaw et al., 2008a), Tanzania | Surveys (n = 240)  Year of study = 2004 | Evaluated the adoption and impact of farmers’ and market-preferred and disease-resistant pigeonpea varieties that were developed and promoted in semi-arid Tanzania. | The data from northern Tanzania shows that **several households were constrained from adopting new varieties due to limited seed access**. Some potentially adopting farmers were prevented from growing new varieties due to a lack of access to seed. The results show that the new pigeonpea varieties improve household incomes by up to 80%. Despite the benefits of improved varieties, many farmers did not adopt the disease-resistant varieties mainly due to a lack of access to the seed and agronomic information on the new cultivars. This study shows that although the exchange of seeds among farmers is important in improving access to new seeds, it is not sufficient to distribute the varieties widely and efficiently. | There is an urgent need to strengthen rural institutions and involve them in addressing market failures in both seed and output markets in areas with limited market infrastructure.  Promotion of improved varieties, combined with better local availability of improved seeds at reasonable prices offers the most promising policy mix to scale adoption. The participation of farmer organizations and the private sector can be expanded through better promotion, seed production, distribution, and marketing strategies. |
| (Tufa et al., 2019), Malawi | Surveys (n = 1237)  Year(s) of study = 2016, 2017 | Examines the impacts on farm productivity and income levels following the adoption of improved soybean varieties and agronomic practices (ISVAPs). | This study found that more than a third of the households involved in this study adopted the ISVAPs. Additionally, it was found that soybean yield and income from crop sales were significantly higher for those that adopted the practices than for those that did not. Farmers who adopted the ISVAPs had on average a 61% yield increase and a 53% income increase. | This study suggests that ISVAPs should be scaled further, and adoption should be encouraged in order to increase the livelihood benefits associated with these practices. |
| (Verkaart et al., 2017), Ethiopia | Surveys (n = 700, 661, 631)  Year(s) of study = 2007/07, 2009/10, 2013/14 | Determined the impact of the adoption of improved chickpea varieties on the welfare of Ethiopian smallholders. | **The adoption of improved chickpeas was found to significantly increase household income**. The only group whom the adoption of the improved variety did not benefit were the largest landholders. Increasing access to improved varieties of chickpeas appears to hold potential for improving the livelihoods of Ethiopian smallholders. | Further analysis of the results of this study to determine the main drivers of adoption could be used to inform future studies and future scaling of improved chickpeas. It was found that seed systems must be developed in order to increase access to improved seeds. |
| (Waldman et al., 2017), Malawi | Surveys (n = 488)  Year of study = 2014 | To determine the trade-offs between annual and perennial pigeonpea production for smallholders. | One main trade-off reported by farmers in relation to perennial pigeonpea when intercropped with maize is the competition with maize after several years of production however other farmers find maize and perennial pigeon pea to grow well in an intercrop system. Although maize yield is still the factor of most importance to farmers in a maize-pigeon pea intercrop, the perennial pigeon has seen high demand due to its positive effect on soil quality and pigeonpea grain yield. | Future research in this area should look further into the benefits of perennial pigeonpea such as reduced labor and seed input requirements. Research into breeding perennial crops for traits such as early flowering could utilize the benefits of perenniality while also reducing the risk of yield losses due to depredation. |
| (Zulu et al., 2018), Malawi | Focus groups (n = 117), key informant interviews (n = 24), participatory rural appraisal  Year of study = 2017 | Explores the constraints to pigeonpea adoption and identifies approaches used to prevent livestock damage to pigeonpea including Community Based Livestock Management (CBLM). | The results of this study suggest that smallholders are willing to partake in community livestock-management approaches in order to minimize damage to their pigeon pea crops when the economic benefits of adoption are greater than the costs. Damage from livestock was the largest constraint to pigeonpea adoption in newly adopted sites. Several of the crop protection approaches were found to be time-consuming, ineffective, and costly. CBLM controlled damage to crops by livestock and was moderately effective over the course of the year. | This study suggests that CBLM should be further researched and determinants of the successes or failures of CBLM for crop protection should be identified in order to scale this up as a strategy. |
| (Barnes et al., 2021), Kenya, Democratic Republic of Congo | Structured questionnaire (n = 274)  Year(s) of study = 2018-2022 | Explores the drivers behind the adoption of legumes by developing an indicator of household legume cultivation (HLC) from a bespoke survey of 274 small-scale farm households. | Find a range of intensities across sites and farms, indicating the limited influence of agro-ecological zones and formal institutions on uptake. There was some commonality in drivers across sites, though age, income, and gender have positive but very marginal effects. Farm households with more intense legume cultivation were less driven by commercial growth objectives and had limited access to markets. There was little interest in expanding farm area which reflects the lack of assets available to these farmers and, therefore, promotes the use of legumes in providing home nutrition or supporting farm fertility and provision of livestock feed. | Overcoming constraints to increasing the use of legumes should be a significant component of local and international agricultural intervention as countries experience increasing environmental and social pressures and the need to commercialize as farming develops. |
| (Jelliffe et al., 2018), Uganda | Surveys (n = 480)  Year(s) of study = 2004, 2014 | To investigate the sustainability of a farmer-led program for the distribution and multiplication of high-yielding varieties (HYVs) of groundnut. | 21% more land was planted with HYVs of groundnut by households participating in the study than by controls. The mean adoption of HYVs by neighbors of participating farmers was found to be higher than non-neighbors which shows some diffusion of program benefits from participants to their neighbors. **Due to seed-saving by farmers, the yields of HYVs decreased over time to eventually be below those of landrace varieties**. | This study indicates the need for greater levels of education to increase the sustainability of projects such as this and to ensure greater food security for households. |
| (Mahama et al., 2020), Ghana | Surveys (n = 300)  Year of study = 2018 | To find the level of adoption intensity of several soybean production practices. | Farmers involved in the study typically adopted 50% of the technologies to which they were introduced. Factors that positively influence adoption include age, extension services received, information access through mass media (radio), and perceptions of soybean production being a risky practice. | The recommendations from this study are that there should be increased education through extension services surrounding the benefits of sustainable soybean production practices. When educating farmers on new technologies demonstration farms giving opportunities for practical training are very important. |
| (Makate et al., 2018), Mozambique | Surveys (n = 332)  Year of study = 2015 | To identify various types of farm households based on socioeconomic characteristics and how this related to their adoption of various bean production, management, and marketing practices. | The results of this study demonstrate that there are several socioeconomic factors that can relate to the adoption of innovative bean farming practices. Farm types identified were: 1) female landowners with small farms (29.52%), 2) educated farmers with access to credit (6.63%), 3) wealthy male farmers with low education and large farms (8.73%), 4) young, poor male farmers with low levels of experience (6.33%) and 5) female farmers with significant experience and high labor capacities (8.43%). Each of these farm types showed different patterns of adoption of innovative bean farming practices. | This study suggests that approaches are promoted by policymakers that aim to increase the adoption of innovative practices of bean farming, management, and marketing and that policies such as this consider the many different household types. The 5 household types found in this study can be an initial suggestion of farm types in Mozambique. |
| (Grabowski et al., 2019), Malawi | Focus groups, interviews (n = 488), crop model created  Year of study = 2014 | To investigate the adoption and diffusion opportunities for the perennial management of pigeonpea in maize-dominant systems in environments characterized by high rainfall variability. | Perennial pigeonpea cropping was shown the be beneficial on farms where maize yield and pigeonpea biomass values had remained constant for some time. The practice was adopted best in areas where dry growing seasons highlighted the benefits associated with perennial systems. Social pressures to conform to typical cropping systems reduced adoption potential. | New practices may not receive the level of adoption initially expected from research trials as climate variability can impact farmers' ability to determine the effects of new technology and this may result in slightly lower adoption. Farmer-to-farmer extension services in combination with access to expert support are more likely to create trust in a new technology and encourage adoption. Dis-adopters were also shown to have a high potential to reduce adoption levels by damaging trust in the technology. |

**Supplementary Table 5. Research studies that focused on the adoption of underutilized crops.**

| Author, Location | Methodology | Objectives | Key findings | Recommendations |
| --- | --- | --- | --- | --- |
| (Abay et al., 2008), Ethiopia | Surveys (n = 240), focus groups, and informal discussions  Year of study = 2008 | To develop a greater understanding of local barley varieties to identify farmers' preferences in relation to them and to find the perception of farmers of the values, constraints, and opportunities of producing local barley varieties | The selection of varieties and seeds by farmers was impacted by various features of each variety and of the environment.  Male farmers more often chose the variety which would be produced. Female farmers carried out seed storage and processing. | The combination of local and scientific knowledge of the varietal selection and breeding process could result in the development of improved varieties which are both beneficial for and preferred by farmers. These varieties would have significant potential for adoption in this region. |
| (Boadu et al., 2018), Ghana | Surveys (n = 380)  Year of study = 2015 | Analyzed the effect of farmers’ thoughts about the quality of seed yam that was generally used for cultivation and how this impacted their preferences for certified seed yam. | The results of this study found that it is the feeling of farmers that certified seed yams would increase yield by reducing losses due to pests/diseases. If the quality of the improved seed is assured farmers said they would be willing to pay up to 26% more for improved seed. Perceptions about improved seed yam and about the quality of the yam that was produced were impacted by the level of education, years of farming experience, income, and level of extension services received. | Recommendations are increasing access to extension services which focus on seed quality assessment and additional agronomic practices to increase farmer education. Improving farmer's ability to assess the quality of their crops would help improve their yields and impact their perceptions of improved seed. |
| (Kaliba and Mazvimavi, 2021), Tanzania | Survey (n = 822)  Year(s) of study = 2013, 2014 | To identify how the adoption of improved sorghum varieties impacts the welfare of smallholder households. | All welfare indicators used in this study were significantly improved through the adoption of improved varieties. Food insecurity and poverty levels can both be reduced by adopting improved crop varieties. The impacts are different across the regions studied. Adoption of the improved varieties and the impacts they have are increased when farmers are operating in a better-built environment. | Specific training on improved varieties for the poorest farmers could have significantly positive results. It was found the environment farmers work in (e.g., marketing services) also had positive impacts along with the use of improved varieties. |

**Supplementary Table 6. Papers that focus on barriers to scaling legume seed systems.**

| Author, Location | Methodology | Objectives | Key findings | Recommendations |
| --- | --- | --- | --- | --- |
| (Ali and Awade, 2019), Togo | Structured questionnaire (n = 500)  Year of study = 2018 | To evaluate how credit constraints affect the welfare of subsistence soybean farmers in Togo. | The main factors identified to determine full access to credit are the age of the farmer, membership to the soybean organization or to a recognized NGO, or the production of cotton or cashew. **Men have greater access to the full amount of credit available than women farmers**. Completion of education and extension programs both have a positive impact on the welfare of smallholders. Female farmers can also improve their welfare compared to male farmers by increasing their land cultivation and using intercropping as a method of conservation agriculture. Access to the full amount of available credit increases the production of soybean soybeans and increases the revenue achieved by smallholders by 1.32%, compared to a lack of this credit. | The results found in this study emphasize considering gender and the varying levels of access male and female farmers have when considering agricultural credit for soybean farmers and how access to this credit affects the wellbeing of farmers and their households. |
| (Anago et al., 2021), Benin | Interviews (n = 606)  Year(s) of study = 2017, 2018, 2019 | Understanding the factors leading to yield gaps in cowpea cultivation. | Results showed that avg. cowpea grain yields in farmers’ fields were low and yield gaps were seen between fields in the north and the south of Benin with the fields in the north experiencing the lowest yields. The study identified that the low yields are because of poor soil quality and nutrient content as well as suboptimal crop management practices. **The greatest limiting factors of yield are pests, diseases, and decreasing soil nutrient levels**. Due to this, yields could be increased using inputs such as fertilizer, insecticide, pesticide, and improvement of soil quality. | Future research in this area based on providing specific input recommendations would benefit cowpea farmers and help reduce yield gaps. |
| (Boni et al., 2021), Tanzania | 180 groundnut and 200 maize samples were collected from 9 and 10 districts, respectively.  Year of study = 2012 | To find levels of aflatoxin contamination in maize and groundnuts across Tanzania. | Aflatoxin levels ranged from 92 to 100% for groundnuts and 10 to 80% for maize and aflatoxin was found in samples taken from every district. 75% of the farmers whose samples were tested were unaware of aflatoxins or the negative health impacts of consuming contaminated products. This study shows that across Tanzania aflatoxin contamination of staple crops is widespread and this poses risks to humans due to dietary preferences for crops that are widely contaminated. | The issue of aflatoxin must be communicated to both farmers and consumers to increase awareness and reduce the risk to consumers. |
| (Gebreyes et al., 2021), Ethiopia | Focus groups, key informant interviews (n = 10), document analysis  Year of study = 2012 | Contributes to research on scaling and tackling major scaling constraints | The results of this research find that scaling of agricultural innovations must consider both technical requirements and social dynamics related to scaling targets and the social dynamics of stakeholders. Additionally, it is essential to understand that scaling is a complex, non-linear process. Scaling often depends on relationships and trust developed with key stakeholders and groups and involves monitoring processes and learning from successes/failures. Considering all the above points, scaling approaches must be dynamic, gradual, and reflective. | In order for scaling to be most effective the approaches taken must be inherently flexible and willing to adjust according to unforeseen obstacles or opportunities that arise. |
| (Girma et al., 2011), Ethiopia | Survey (n = 100)  Year of study = 2001 | To develop a greater understanding of local knowledge of the toxicity of grass pea and their consumption as a result. | Various approaches were employed to avoid lathyrism including avoidance of grass pea consumption in the form suspected to cause this issue. Grass pea is also combined with other crops and various processing and detoxification methods were used. | Further research in this area could focus on breeding grass pea cultivars with lower levels of b-ODAP to increase safety and could assist in developing indigenous processing methods to increase effectiveness. |
| (Misiko, 2013), Kenya | Survey (n = 300), Key informant interviews (n = 15), Participatory learning trials (n = 6), Focus group discussions (n = 6)  Year of study = 2008 | Illustrates obstacles involved in Participatory Varietal Selection (PVS). | Findings reveal significant difficulties in PVS that resulted from unexpected factors such as new crop disease and floods. Spoiler factors reduced the sustainability of participatory gains. Besides, learning during critical field stages was thin due to decreases in participation. For instance, an average of only 8% (total = 1488) consistently participated in each key stage of PVS learning trials in western Kenya. | PVS must be anchored within an integrated knowledge exchange system that addresses challenges within the interaction of smallholder social, spatial, and temporal contexts. |
| (Mugisha et al., 2019), Uganda | Survey (n = 228)  Year of study = 2018 | To identify the reason for the yield gap between male and female groundnut producers. | Improved groundnut varieties had a 63% yield gap and local varieties had a 44% yield gap, with male farmers achieving higher yields than female farmers. Improved seed increased yields of female farmers but not male farmers. Over 70% of the yield gap of both improved and local varieties can be accounted for by the combination of male advantage and female disadvantage. There are differences in labour use between male and female farmers and the yield gaps are caused in part using different varieties. | The yield gap could be reduced by increasing access to inputs for female farmers. Policies and input schemes can help increase the productivity of women’s crops and result in increased food security and income. |
| (Mulesa and Westengen, 2020), Ethiopia | Key informant interviews (n = 26)  Year(s) of study = 2017-2018 | Identified the reason that some countries have restrictive access governance regimes, using Ethiopia as an example. | Three factors are determined which may offer an explanation for Ethiopia’s policy, 1) how Ethiopia’s reputation as a place of high biodiversity influences Ethiopian cultural identity, 2) the economic importance of agriculture based on PGFRA originating in Ethiopia, 3) the influence of movements which promote the rights of the farmer in order to tackle strict intellectual property laws and which highlight on-farm PGRFA management as an option for ex situ conservation and for the strengthening of the formal seed system. | Future research on the governances and policies relating to PGRFA must consider the national context and the historical, social, political, and institutional factors at play within each country that may influence policy. |
| (Nchanji and Lutomia, 2021), Burkina Faso, Burundi, Cameroon, DR Congo, Eswatini, Kenya, Lesotho, Madagascar, Tanzania, Uganda, Zambia, Zimbabwe | Surveys (n = 291)  Year of study = 2020 | Identified the impacts that the COVID-19 pandemic had on bean production and food security. | The research conducted in this study shows that COVID-19 generated some major challenges to bean production. These challenges included limited access to seed, inputs, labor, and finance. It was also suggested that the challenges that arose because of the pandemic may reverse gains made towards Sustainable Development Goals (SDGs) 1 and 2. | Direct investment should be supplied by governments to develop input supply systems and shorter food supply chains. It is proposed that this is done through digital access and food delivery. |
| (Nchanji et al., 2021a), Kenya, Uganda, Tanzania, DR Congo, Burundi, Zambia, Zimbabwe, Mozambique, Cameroon | Survey (n = 856)  Year of study = 2020 | To understand the immediate impacts on common bean production systems of the COVID-19 pandemic and how these may impact food security. | It was found that the pandemic and the related government restrictions influenced access to and cost of inputs. Also, the distribution and consumption of beans were impacted by these restrictions. Southern Africa immediately faced severe effects of the pandemic with Central Africa impacted slightly less. Food consumption patterns were negatively impacted due to production and distribution challenges. | Input subsidy schemes that currently exist need to be strengthened in order to supply inputs to the poorest farmers in times of crisis such as the pandemic. Agriculture must be viewed as an essential service in order to avoid rising levels of food insecurity. Collaboration between public and private bodies is required in order to identify seed sources to be used when production is constrained. Access to credit must also be increased in order to increase the resilience of all stakeholders within the value chain. Small and developing seed enterprises should be supported in developing approaches that increase resilience such as the establishment of digital business models or online sales. Mechanisms should also be put in place to keep markets open safely during the pandemic and to maintain food distribution. |
| (Sinare et al., 2021), Burkina Faso | Interviews (n = 124)  Year(s) of study = 2015-2017 | To explore groundnut production and its constraints. | It was found through this research that groundnut farming is highly vulnerable to climate change. Production of groundnut is significantly influenced by gender, 48.39% of women are involved in groundnut production but have less access to land and resources than men, which has resulted in a gender yield gap, with men yielding higher groundnut yields than women. The use of improved varieties was closely linked to access to extension services. The main barriers to production differed throughout the region however, similar constraints were reported in all areas. **The main constraints included a lack of improved varieties, credit, and tools, additionally, the cost of seed and inputs was too high for many farmers, and drought and disease negatively affected crops also**. | In order to promote and increase levels of groundnut production new technologies and practices are required. Breeding programs must be strengthened in order to develop improved varieties and must consider farmer trait preferences and needs in order to enhance adoption. Seed companies and extension services must work collaboratively to multiply and distribute seeds more effectively. |
| (Tongruksawattana and Wainaina, 2019), Kenya | Surveys (n = 613)  Year of study = 2011 | Determined the main climate shocks that impact maize-legume farming and how farmers in Kenya adapt to these shocks. | Adaptive action is not always taken by farmers and whether it is taken will be influenced by food and income levels following the shock event. Decisions to undertake an adaptation strategy are not found to be influenced by poverty or food insecurity. Female-headed households are more likely to take adaptation measures in response to excessive rainfall than male-headed households. Factors that negatively influence the likelihood of adaptation are farm size, level of education, household size, access to credit, and high temperatures. | Households that are often most vulnerable to climate shocks are female-headed, food insecure, and poor households so policy should aim to support these households in undertaking adaptation measures. Increased assets and access to technology could help these households adapt. |
| (Branca et al., 2021), Malawi | Comprehensive Value Chain (VC) Map of cereal-legume production, a SWOT exercise, surveys (n = 340), policy analysis  Year of study = 2017 | To develop a greater understanding of the VC. Based on a policy analysis, our objectives are to identify the ways in which the value chain interacts with policy. | Limited access to land, technology, and inputs, knowledge of how the functions of the market, insufficient access to credit and extension services, combined with more general problems of poor infrastructures, often stand in the way of smallholder farmers from accessing profitable market opportunities. Inadequate financial capacity, an inefficient public extension services system, limited involvement of the private sector in providing extension services, poorly developed managerial practices within cooperatives, poor market coordination and development, and limited engagement of investors in market infrastructure are the main factors that are identified as limiting the effectiveness of policy supporting VC inclusion. | Contradictions across policies show the need to harmonize and improve existing policies to promote the development of smallholder-friendly value chains through (i) A review of current legal frameworks in an effort to improve them and remove inadequacies (ii) increasing the budget allocation accompanied by proper accountability measures to ensure judicious use of the funds which target policies (iii) the provision appropriate, qualified staff to implement the policies; and (iv) promoting the policies among communities and making smallholders aware of how they apply to them in order to ensure adherence |

**Supplementary Table 7. Research papers focused on options for scaling legume seed systems.**

| Author, Location | Methodology | Objectives | Key findings | Recommendations |
| --- | --- | --- | --- | --- |
| (Akinyemi et al., 2003), Nigeria | A split-plot design with four replications  Year(s) of study = 2000-2001 | To determine the performance of cowpeas cultivated using three different tillage systems. | Results show that the ridge tillage system gave the highest yield and the best economic gains for farmers. Differences between the tillage systems across variables of plant height and fresh weight of pods per plant were significant. In all other parameters measured (leaf number per plant, leaf area per plant, and weight of straw), significant differences were not seen. | This study determined that ridge tillage systems gave the highest yield, yield attributes, and economic benefits for farmers |
| (Akpo et al., 2020), Ethiopia | Key informant interviews (n = 4)  Year(s) of study = 2018 - 2019 | To analyze the experiences of seed producers within the informal system who moved into formal private seed companies. This was done in order to understand how well these farmers were supported in their efforts to become formal, commercial producers of seed. | The seed enterprises studied began with approx. USD300 and at the completion of the study had over tenfold the capital they had when beginning the study. Farmers received a range of supports, such as training in the production of quality seed, marketing, partnerships, and value chain development training. Also, they had access to infrastructures, from extension workers, research centers, various NGOs, and other seed companies such as major public seed enterprises and agro-dealers. The seed enterprises produce pre-basic, basic, and certified seeds of a range of crops which are then supplied directly to farmers, institutions, and agro-dealers. | To develop better systems of production and delivery of seed in SSA seed enterprises which currently exist solely in the informal system need to be supported and trained in the production of certified seed, allowing them to transition into the formal system thus increasing incomes and providing higher quality seed to their communities. |
| (Anders et al., 2020), Malawi | Field experiments, Focus group discussions, Key informant interviews, Demonstration trials, Surveys (n = 366), field measurements  Year(s) of study = 2014, 2016 | To investigate the adoption of grain-legume technologies (GLT) on maize-based systems by smallholders in Malawi. This was done following four years of intentional on-farm experimentation and there was a focus on understanding farmers' why levels of adoption were still low at this point. | The research identified that low levels of adoption of GLT persist. maize-based system was preferred by farmers over the GLT used in this study. It was seen by farmers that the maize-based system was better for the food security of their households. Although labor requirements of GLT were less than the traditional maize systems overall there was still a preference for traditional systems as food security was the focus for these farmers. Increased labour during times such as sowing periods could not be sacrificed by farmers in order to tend to legume crops therefore farmers preferred to just maintain their maize-based systems. | Development of GLT should consider that for many farmers increased labour will influence them against adoption of the GLT.  When developing technologies in the future researchers must consider that there are factors that can deter adoption outside of biophysical and farmer preferences. Labor demands of other crops which would be sacrificed in order to cultivate legumes can be a deterrent to the adoption of the GLT particularly if these are time- or yield-sensitive. |
| (Baoua et al., 2012), Niger | Experimental units (n = 36) of each treatment were set up and checked every 30 days.  Year of study = 2009 | To determine the method of cowpea storage that is the most effective in reducing infestation.  Methods tested were hermetic (PICS) bags, the use of ash, sand, leaves, and stems of *B. senegalensis* and phostoxin. | The phostoxin and solar disinfestation treatments gave the lowest amounts of infestation. The control and *B. senegalensis* treatments showed 8 - 9 times more insect infestation than the most effective treatments. The ash and sand treatments were more effective than the control but significantly less effective than the phostoxin treatment. Hermetic bagging and solar disinfestation gave similar results to phostoxin treatment. Losses of 51.58% were observed in the control and 60.94% were observed in *B. senegalensis* treatments. These were the highest losses seen across all treatments. | Phostoxin and hermetic bagging were significantly better methods of storage when protecting against pest infestation of cowpeas regardless of bag size. Despite its effectiveness, it must be noted that phostoxin has a level of toxicity and as a result, bags containing this treatment must be stored in a location away from humans and animals. The results found in this study can inform extension officers who can then pass on suggestions to farmers about which treatments are best for minimizing their post-harvest losses. |
| (Deu et al., 2014), Mali | Surveys (n= 22) randomized complete block trials with four replications.  Year of study = 2009 | Explores how effective farmers’ methods for preserving varietal seed purity and genetic integrity of an improved variety. | Introgression and contamination were minimized most effectively when (1) farmers had received specific training in seed production, (2) they could take advantage of isolated fields and (3) they could practice true-to-type panicle selection. | Education on the impacts of seed saving and management, particularly for farmers with central roles in seed networks could help increase seed security within informal seed systems and could provide access to improved varieties for farmers who are not served by the formal system. |
| (Dzanja et al., 2017), Malawi | Interviews with companies (n = 9)  Year(s) of study = 2010,2011 | Looks at the option of using markets to promote multipurpose legume cropping systems by creating an increased demand for legumes in the future. | Soybean had the greatest chances of growth, followed by pigeonpea, groundnut, and cowpea. **The main barrier to growth is aflatoxin contamination in groundnuts which points to the need to manage aflatoxin levels**. Female farmers could increase their income by using processing technologies as they are currently confined to retail markets. | Female entrepreneurs should be supported in developing their businesses. Value addition is a method through which women can increase their income from their products. All actors in the value chain including farmers, traders, processors, and exporters must work together to reduce aflatoxin levels in their products. |
| (Franke et al., 2014), Malawi | Survey (n = 77), model developed.  Year of study = 2010 | Assessment based on forecasts of the effect of grain legume adoption on different types of farms and finds niches for grain legume production in Malawi | **Production of legumes can positively impact food self-sufficiency for poor farmers if the crop is treated with P fertilizer and inoculation for soybeans**. Due to the low ability to invest in poor farmers, these requirements may limit the adoption of legumes amongst low-resource farmers. | This study finds that there is potential for legumes to be a cash crop, but more so amongst farmers with medium to high resources. For poor farmers legumes have the potential to improve food self-sufficiency but these farmers are limited by their ability to invest in legume-based technologies. |
| (Gama et al., 2018), Malawi | Surveys (n = 489)  Year of study = 2018 | Examines consumption of peanuts and the factors that influence peanut consumption in Malawi in order to identify consumer willingness to try new peanut-based foods. | 70.4% consumed peanuts at least three times a week. Preferences for various peanut products, peanut consumption, and willingness to try new products were significantly affected by demographic and socioeconomic factors. Females preferred peanut flour more than men. Peanut butter was preferred by younger consumers. Education influenced peanut consumption, consumers with high school education and below ate peanuts significantly more often than those with education past high school. Men were more likely to try new foods than women and those with higher levels of education were more likely to try new foods than those without. | At present the peanut-based food products on the market in Malawi are limited. Producers of new and innovative food products must identify ways to overcome the socioeconomic barriers to consumers trying new food products. |
| (Gumma et al., 2019), Malawi | The spatial distribution of cropland areas was mapped.  Year(s) of study = 2010–2011, 2016–2017 | To monitor cropland areas in Malawi using MODIS 250 m 16-day time series data in order to identify the distribution of pigeonpea and groundnut cropping, following the release of new varieties. | Pigeon pea is largely produced as an intercrop with groundnut in the southern dry districts of Mulanje, Phalombe, Chiradzulu, Blantyre, and Mwanza and parts of Balaka and Chikwawa., and is intercropped with sorghum in the Mzimba district. This study shows that the area planted with pigeon peas had increased by 75,000 ha (29%) during the time studied. | Through the use of these mapping technologies, appropriate locations can be found in which to demonstrate improved management practices and promote improved varieties in order to scale up and scale out these practices/crops most effectively. |
| (Gwenambira-Mwika et al., 2021), Malawi | Various research sites (n = 6) were tested.  Year(s) of study = 2015-2019 | To examine various groundnut pigeon pea production systems and measure productivity, economic performance, and total N accumulation. | Rotations of pigeonpea and maize produced the greatest biomass levels but economic and agronomic returns during the legume phase were only moderate. **Results found that in hot, dry environments maize yields were increased most when in a rotation with groundnut**. | The systems tested in this study were shown to be a good method of increasing biological nitrogen fixation at low costs while also resulting in multiple grain harvests throughout the year. These systems are effective across a range of environments and are therefore a good option for ecological intensification. |
| (Haileyesus and Mekuriaw, 2021), Ethiopia | Surveys (n = 203)  Year of study = 2015 | Identifies the effect of wheat chickpea double cropping adoption on yield and farm income of rural smallholders in Oromia Region, Ethiopia | This research shows that smallholder households that adopted wheat-chickpea double cropping systems were significantly positively impacted (yield and income) compared to households that did not adopt this system. | In order to increase adoption and scale these cropping systems barriers and enablers to adoption must be identified and addressed. Access to improved seeds, specific extension services on double cropping, and access to fertilizer are all areas that should be addressed in order to encourage adoption. |
| (Hillyer et al., 2006), Namibia | Interviews (n = 6), maps created, Soil samples taken  Year(s) of study = 1998-2001 | To explore local knowledge of soil and land management in relation to legume cultivation. Land ownership patterns were also explored. | This study showed that smallholders have a practical and in-depth knowledge of how best to manage their land. Traditional methods include information about land type, fertility, and management under various levels of rainfall. T**his study shows the importance of NGOs and extension officers engaging with indigenous communities** when attempting to select the best interventions for the location. | The approach used in this study to gain an understanding of indigenous knowledge and use it to inform development projects could be applied across SSA. |
| (Hoffmann et al., 2018), South Africa | Tested crop model against field trial data and performed crop model simulation runs for all cultivated land in Limpopo.  Year(s) of study = 2013/14, 2014/15, 2015/16 | To explore the effect of the cultivars and planting dates on groundnut yield | Field trials showed that early planting was better than later plantings, irrespective of the weather year. For the generally drier regions of Limpopo, a cultivar with a shorter growth duration would indeed be more productive in *El Niño* years. It showed that such season-specific adaptation would increase yields by 2.9% for Limpopo. | This study demonstrated that management recommendations at the provincial scale explicitly need to consider in more detail a location-specific and season-specific approach. |
| (Kaizzi et al., 2012), Uganda | Fertilizer response trials were conducted for three legume crops - bean, soybean, and groundnut.  Year(s) of study = 2009-2010 | To determine the optimum combinations of crops/ nutrients/ application rates to give the greatest returns to fertilize use and greatest benefit: cost ratio. The crops studied were beans, soybeans, and groundnut. | Nitrogen fertilizer applied to beans gave the best benefit: cost ratio, followed by P fertilizer on soybean or groundnut, then P used with beans, and lastly, K on groundnut or soybean. | The results of this study allow optimal investment by smallholders, by providing information on the best fertilizer and management practices for specific crops. This specific information can provide smallholders with the greatest return on their investment in fertilizers and is important when the resources and capital required to buy inputs are low. |
| (Kilwinger et al., 2021), Rwanda | Interviews (n = 390)  Year of study = 2019 | To develop an improved understanding of various farm typologies in order to inform the development of specific seed business models. | Results found that farmers who produce seed for commercial use have more access to formal sources of seed. Despite this improved level of access, most **farmers of all types obtained new varieties and quality seeds from informal sources**. Cash purchases were made more by wealthier farmers and tended to be single investments in order to obtain a new crop variety. | This study suggests that when developing seed business models, it is essential to consider seed sourcing practices, farmers' differences, how willing farmers are to pay for seed, cost-benefits, and the role of seed degeneration. Creating seed business models that consider these differences has significant potential however various models must be carefully developed so that they do not contrast with others. |
| (Koona et al., 2007), Cameroon | Two storage method treatments were studied.  Year of study = 2005 | To identify which of the two treatment methods were most effective in reducing damage by bruchid insect pests. One method used bags impregnated with aqueous extracts from two plants with insecticidal properties and another used leaf powders applied directly to the seed. | The seed treatment method was the most effective, followed by the bag treatment method, followed by the control. | Although the seed treatment was the best option the bag treatment option could also be considered particularly over long periods of time, allowing farmers to store seed and sell when market prices are high, or alternatively spread revenue throughout the year. |
| (Majili et al., 2020), Tanzania | Surveys (n = 303), Focus groups (n = 60)  Year(s) of study = 2019, 2020 | To identify the consumer preferences and levels of consumption for pigeon pea. This information is to be used to inform the development of pigeon pea-based food products aiming to improve nutrition and income. | During the harvesting season, 80g/person/day of pigeon pea were consumed and during the lean season, 18g/person/day were consumed. Most farmers consume local varieties of pigeonpea, 84% were eaten as stews and there are 5 recipes for pigeon pea in the region studied. Factors that influenced the level and preference of pigeon pea consumption were availability, taste, source of income, and familiarity with the variety. | Consumption of pigeon peas could be increased as there are currently constraints to consumption. New and innovative concepts for shelf-stable products that meet the preferences of farmers could lead to higher levels of consumption. |
| (Manda et al., 2020), Nigeria | Surveys (n = 1,525)  Year of study = 2017 | Investigate the effect of market participation on food security and income for households producing cowpeas. | The research found that participation in a market had a significantly positive effect on cowpea-producing households. **Household food security and income both increased with market participation.** In addition to selling in markets, selling to traders (both rural and urban) also positively impacted food security, food expenditure, and income. | This study suggests that there is a necessity for policy and public infrastructure improvement to allow for greater levels of market participation. Construction and maintenance of rural roads could greatly improve opportunities and welfare for rural farmers. |
| (Martey et al., 2021), Ghana | Survey (n = 320)  Year of study = 2019 | Determines the impacts on productivity, adoption, and income of cowpea-producing smallholders’ involvement in a program focussing on novel comprehensive agricultural training (CATP). | The study found that farmers who were involved in the CATP had 75% greater levels of adoption, 15% greater productivity, and 24% greater income due to cowpea, in comparison to mean levels. | It is suggested that projects aiming to improve involvement in CATP must continue to promote the training past just the project period in order to achieve project goals. Benefits to the income and welfare of farmers as a result of their involvement in CATP can be increased by increasing awareness and removing barriers to participation in such programs. |
| (Misiko et al., 2008), Kenya | Plots were managed according to farmers’ practices and evaluated using participatory monitoring and evaluation approaches.  Year of study = 2004 | To understand the process of selecting soybean varieties by smallholders for soil fertility management in western Kenya. | Farmers’ selection criteria fell into three categories relating to yield, appearance, and labor requirements. Soil fertility was not the primary aim when farmers were selecting preferred traits. This created a challenge to embed the new varieties within the local farming systems for soil fertility improvement. | We propose research activities involving farmers to strengthen farmer experimentation skills, their understanding of N addition, and the role of P in soil fertility. |
| (Mkhize et al., 2022), South Africa | Questionnaires (n = 120)  Year(s) of study = 2015-2017 | The introduction of grain legumes, determining the yield of grain legumes, increasing consumption of legumes, and educating farmers on food production maximizes the nutrition of the crop. | Results showed significant differences between the legume varieties studied. Yields of crops produced by women were significantly higher than those produced by men. Over the course of the study, legume consumption was found to have increased from 1 time per week to 2-3 times per week. | Projects focusing on nutrition should examine policies that control the import and export of legume varieties that are not grown locally in order to increase access to a wider variety of legume crops. |
| (Mligo and Craufurd, 2005), Tanzania | Ten cultivars were planted at six locations, and measurements were taken.  Year(s) of study = 1994, 1995 | To measure biomass (BY) and seed (SY) yield of a set of phenologically diverse pigeonpea varieties to determine their adaptation to various environments in Tanzania | Extra-early cultivars such as ICPL 86005 also showed potential, particularly for short-season environments. | This type of simple but robust analysis would be appropriate in many less developed countries, where the analysis and collection of more in-depth physiological data sets are often difficult and may help in identifying better-adapted genotypes of other crops. It would also be useful to analyze longer-term weather data to establish a target mean crop duration, as well as to identify target environments. |
| (Mohammed et al., 2020), Nigeria | Survey (n = 420)  Year of study = 2017 | Examined the knowledge of farmers about fertilizers and determined factors that influence the use of P-based fertilizers. | 80% of farmers involved in the study were aware of the benefits of using fertilizers in order to increase yield and also were capable of identifying when a crop was lacking certain nutrients, however, despite this only 10% used P-based fertilizers and 11% used N, P and K fertilizers for cowpeas. **The high cost, low availability of fertilizers, and lack of knowledge regarding the need for fertilizers were constraints to farmers' use**. It was found that many farmers did not believe that cowpea crops required fertilizers. | The results of this study provide information that has the potential to help increase the productivity levels of cowpea crops. Appropriate use of fertilizers can increase yields and reduce the need for cowpea imports in Nigeria. |
| (Nassary et al., 2020), Tanzania | A randomized complete block design (RCBD) was conducted.  Year(s) of study = 2015-2017 | To understand whether smallholder informal maize monocrops could be replaced by rotations including common bean. | Research from this study showed that in both the short and long rainy seasons bean gave significantly higher grain yield. | This study suggests that the use of an intercrop of maize and common bean resulted in higher grain yields and therefore increased sustainable food production for smallholders. |
| (Nord et al., 2021), Tanzania | Surveys (n = 413), Focus groups (n = 5), Key stakeholder interviews (n = 12).  Year of study = 2017 | Explores the various agricultural practices used by smallholders, particularly in maize-legume systems. | It was found that legume farming practices were very specific to location. Additionally, analysis of suggestions offered by extension services shows that varieties, inputs, and spacing used by smallholders were not considered. | Extension services must consider the local context when offering recommendations for legume farming systems in order to best support farmer adaptation. |
| (Périnelle et al., 2021), Burkina Faso | Innovative cropping systems (ICSs) were introduced in participatory trials and were evaluated by farmers (n = 70)  Year(s) of study = 2016, 2017 | To develop new legume-based cropping systems using a bottom-up approach. This study utilized the knowledge of local farmers. | This study found that innovative cropping systems that are locally implemented were very appealing to the farmers who attended the field days. Additionally, the approach taken in this study, using participatory methods at two different steps in the process was original and offered a very promising method of designing and introducing new innovations. | In order to investigate this approach further, farmers who attended field days should implement the ICSs on their own farms and make their own adaptations where needed.  This third step in the process will continue to build knowledge, empower farmers, and incorporate legumes into a diverse selection of local cropping systems. |
| (Peter et al., 2017), All of Africa | Climate niches for maize, pigeon peas, and sorghum are identified.  Year of study = 2016 | To locate the marginal agricultural land in Africa by locating the suboptimal conditions for the dominant crop and the optimal conditions for two perennial grains, pigeon pea, and sorghum, as a method for scaling perennial grains. | Low rainfall requirements allow sorghum to grow in locations that pigeon pea cannot, however in general conditions is more optimal for pigeon pea cultivation on approx. 45% of agricultural land across Africa, and the optimal for sorghum on 24%. Approximately 53% of African agricultural lands could benefit from the integration of one of these crops. The potential to improve maize-based farming systems through the integration of perennial crops is considerable, particularly as many of these crops provide multiple ecosystem services. | It is recommended that methods such as the one used in this study are employed by policymakers to target specific areas that would benefit from the implementation of a certain crop. In order to give the greatest results (both for maize yield and the yield of the perennial) it is important to select the best environments for the integration of these perennial crops. |
| (Ronner et al., 2018b), Uganda | Surveys (n = 75)  Year of study = 2014 | To determine the options, barriers, and trade-offs at a farm level in the production of climbing beans for smallholders. | The ex-ante investigation of climbing beans shows that their production had positive impacts on food self-sufficiency and household income but also required increased investment and labor. Few options for climbing bean introduction were found among poor households due to small farm size and lack of ability to make prior investments. Although interest in climbing beans is increasing, their production is limited by a lack of seed and stakes. | Increased access to climbing bean seeds could be achieved through the strengthening of farmer cooperatives. |
| (Rusinamhodzi et al., 2012), Mozambique | Maize-pigeon pea and maize-cowpea intercrops were established at two sites, Focus groups, and interviews (n = 94)  Year(s) of study = 2008-2011 | To examine the farming system and determine whether maize-legume intercropping was an appropriate option to reduce biophysical and socio-economic limitations faced by smallholders in this region. | Farmers found the high productivity and the associated benefits to income of maize-legume intercropping to be attractive despite the increased labor requirements compared to monocrops. Farmers could maintain maize yield by using the within-row cropping method and farmers found this to be an acceptable option. **Legume intercropping can be particularly useful for farmers who do not have access to fertilizer inputs**. In larger farms, the additional labor involved in legume intercropping may be offset through the reduction in land area used. Intercropping also acted as a water management system by offering greater soil cover and more C inputs to the soil allowing greater rainwater infiltration. | This study suggests that maize-legume intercropping can increase resilience by lowering risks of crop failure, improving yield per unit area, and providing greater food security to smallholder households. |
| (Sauer et al., 2018), Zambia | Surveys (n = 7254)  Year(s) of study = 2012, 2015 | To give evidence on how legume adoption affects food security in smallholder households. | The research conducted in this study shows that by adopting rotations of cereals and legumes households saw a statistically significant increase in production of both calories, protein, and crop sales. However, it was also found that there was minimal evidence for cereal-legume intercropping increasing food availability and access among the households studied. | Further research in this area could determine specific cereal-legume intercrop systems that meet farmers' specific needs. It must also be identified whether there are trade-offs or co-benefits involved in the integration of legumes on smallholder farmers which may influence the adoption of these systems. |
| (Sennhenn et al., 2017) Kenya | Legumes were sown at three different plant densities and water treatments.  Year(s) of study = 2012, 2013 | To investigate the potential impacts of climate change and variability on the agricultural potential of common bean, cowpea, and lablab. | The result of this study shows that the risks to crop production due to climate variability are quite high in semi-arid Eastern Kenya and that these risks are increasing. It was found however that the legume crops studied here have potential as solutions to this challenge. Adaptive traits shown by the crops are early flowering and pod and seed set before the onset of drought. The short-season nature of these legumes offers various traits that allow them to have a greater production potential even in drought conditions. This shows that they are quite suitable for production in the areas studied. | The incorporation of these legume crops in areas with challenging environmental conditions offers the potential for farmers to increase the resiliency and productivity of their farming systems. |
| (Senyolo et al., 2018), South Africa | Key informant interviews (n = 32)  Year of study = 2015 | Explores the climate-smart agricultural (CSA) practices that are currently available in South Africa and identifies where and how they are used. | The CSA technologies most used in South Africa are improved seed varieties with drought tolerance and early maturing, rainwater harvesting, and conservation agriculture. Limitations to the adoption of these technologies include the high cost of initial investment, additional labor requirements, and increased management intensity (conservation agriculture and rainwater harvesting). **Greater potential for adoption is seen in drought-tolerant and early maturing seeds as this option is less costly and does not have any increased labor demands**. | This study suggests that increased training and education on CSA practices and the early involvement of farmers in the development of new technologies have the potential to result in greater levels of adoption. |
| (Sidibe et al., 2020), Mali | Surveys (n = 314), diversity fields (n = 150)  Year(s) of study = 2015, 2016, 2017 | Explores the opportunities to use a community biodiversity management approach to increase fonio and Bambara groundnut production through increased producers, yields, and diversity within smallholder farmer communities. | Although the factors directly impacting adoption cannot be clearly defined, those that most likely impact are seed access and increased awareness among the community through seed fairs and community seedbanks. Significant yield increases were not identified. There was an increase in the number of varieties within the community seedbanks from 1-5 varieties of each crop to 11-12 varieties following the study interventions. The diversity of Bambara nut varieties grown by farmers was seen to increase and the number of fonio varieties was seen to decrease in a number of communities. | The results of this study can provide information for future projects aiming to enhance the production of underutilized crop species. Other species such as sorghum, millet, or certain legume crops may face similar barriers, but their production could have significant benefits for smallholders. |
| (Singh et al., 2014), India, Myanmar, Ethiopia, Kenya, Tanzania | CROPGRO-Chickpea model was used to study the impact of climate change and genetic traits on the growth and yield of chickpeas.  Year of study = 2013 | To measure the impact of projected climate change on chickpea yield at study sites, determine benefits on chickpea yield of genetic improvement in relation to duration of crop maturity, yield, drought, and heat tolerance traits | Compared to baseline values climate change was shown to increase chickpea yield at five study sites and to decrease yield at two study sites by 2050. Yield benefits as a result of increased CO_2_ were in the range of 7-20% compared to yields at present. Future changes in temperature and rainfall had either positive or negative effects on yield depending on the site. | This study gave some insights into the traits that will most benefit chickpea yield at various locations. The results can be applied to other regions with similar conditions in order to enhance chickpea yield further. |
| (Snapp and Silim, 2002), Malawi, Kenya | Survey (n = 99)  Year of study = 1998 | Examines farmer participatory research in management options for smallholders in relation to soil fertility and legume intensification. | Longer-duration legumes were shown to produce higher-quality residues than short-duration legume varieties. It is suggested that this is due to a longer time to acquire nutrients, fix nitrogen, and photosynthesize. Additionally, the long varieties had more capacity to recover from drought or pest stress. However, these varieties were also later maturing with higher labour requirements which were not appealing traits for smallholders. High yield, low labor requirements, and early maturing were the traits most preferred by smallholders studied. | Future research should identify varieties that meet the trait preferences of farmers in order to enhance adoption. Short-duration varieties should be complemented with long-duration varieties that can tolerate stresses and improve soil quality. Breeding programs should focus on developing crops that fit well in intercropping systems. |
| (Tabe-Ojong et al., 2021), Cameroon | Surveys (n = 160), interviews  Year of study = 2016 | To identify the main grain legumes for production, consumption, and sale in the region studied. | This study determined that smallholders sell their crops in the market once the family food requirements have been met. Households with female heads are more likely to produce and market legume crops. | Evidence-based policy suggestions are provided by this study which aim to empower female farmers and increase their access to improved seed. It was also noted that indigenous knowledge is very important and therefore informal education about legume production, consumption and sale should be encouraged. |
| (Tsubo et al., 2005), South Africa | Field experiments were carried out at two sites, intercropping maize and legumes.  Year(s) of study = 1996/1997-2002/2003 | To develop a model of cereal-legume intercropping and production under semi-arid conditions. | The model created in this study performed well for yield of both the cereal and legume crops however it was found to be less accurate for leaf and stem biomass predictions. Due to the nature of the root systems of maize and beans, it was found that there is potentially less competition for water between these two crops in an intercropping system. For this reason, the model dealt with light competition rather than water competition. Canopy height was included in the model, but canopy architecture was not. | A basic model was used in this study and could therefore be used as a basis for the development of other cereal-legume intercrop models. |
| (van Loon et al., 2018), Ethiopia, Tanzania, Kenya | Yield-gap analysis for five-grain legume crops (chickpea, common bean, cowpea, groundnut, and pigeonpea)  Year of study = 2018 | To calculate water-limited yield potential (Yw) and yield gaps for the five main legume crops in the region and to determine how narrowing this yield gap could contribute to the food self-sufficiency of this region. | This study determined that the Yg must be closed in order to meet the 2050 legume demand in Kenya. However, it was also found that in Ethiopia intensification of legume production on existing cropland will not be able to reach levels required for self-sufficiency. | There is significant potential for legume production to be improved (for common beans particularly). Future legume demand is predicted to increase due to population growth and increased per capita consumption, therefore the yield gap that exists currently must be closed in order to increase production sustainably and to avoid expansion. |
| (Van Vugt et al., 2017), Malawi | Agronomic trials, random controlled block design (RCBD) with 5 treatments  Year(s) of study = 2009, 2010 | To measure the impacts of several management practices on soybean yields, determine how these technologies benefitted farmers economically, and determine which of these technologies were most preferred by farmers. | The farmers ranked the practices in the following order (1) early planting, (2) plant population, (3) variety choice, (4) compost manure, (5) weeding, (6) inoculant, (7) fertiliser and (8) spraying | This participatory approach revealed that limited access to capital, inputs, and information, the role of soybean, and the perceived risks associated with introducing a new technology were important factors to consider as these were all factors that impacted the success of the study. |
| (David, 2004), Uganda | Case study  Year(s) of study = 1994-1997 | Suggests seed production by FSEs as a method for meeting two goals: to sustainably distribute and promote improved and new crop varieties and to establish a regular source of good quality seed of either local or modern varieties. | While FSEs may provide a sustainable solution to the issue of inadequate seed supply. | Linkages need to be fostered between farmers, researchers, agro-enterprise specialists, NGOs, and the formal seed industry. Seed policy reforms must be implemented and more research systems that consider farmer preferences must be institutionalized. As the model proposed in this study suggests, FSEs must be developed within the context of an integrated seed supply system. This ranges from traditional seed production at the farm level to the formal and commercial seed industry, with each element having well-defined and connected roles |
| (Kuhlmann, 2021), Colombia, India, Vietnam, Kenya, Peru, Brazil, Tanzania, Myanmar | Case studies, key expert consultations  Year of study = 2020 | Addresses knowledge gap surrounding the role of law and regulation in the creation of links between the informal and formal seed systems and creating a more inclusive seed system that is more effectively governed. | This study finds that more flexible regulatory approaches and practices are very important in ensuring there is consistently quality seed in the market and encouraging the introduction of high-quality traditional varieties and those preferred by farmers into the market. | Any model for seed regulation will be context-specific and differences in regulatory systems may indicate potential approaches for improving access, availability, and affordability of quality seed. National and local governments may have the option to adapt policy and regulatory options to local priorities. Flexibility can be integrated into other formal seed systems, reducing gaps between formal and informal seed systems. Local approaches can be highly resilient in the face of market disruptions, such as those caused by the COVID-19 pandemic. |
